# Strain-dependent wetting of graphene

**DOI:** 10.1039/d6sc05377g

**Published:** 2026-10-20

**Authors:** Darren Wayne Lim, Xavier R. Advincula, William C. Witt, Angelos Michaelides, Fabian L. Thiemann, Christoph Schran

**Affiliations:** a Cavendish Laboratory, Department of Physics, University of Cambridge Cambridge, CB3 0HE UK cs2121@cam.ac.uk; b Lennard-Jones Centre, University of Cambridge Trinity Ln Cambridge CB2 1TN UK; c Yusuf Hamied Department of Chemistry, University of Cambridge Lensfield Road Cambridge CB2 1EW UK; d Harvard John A. Paulson School of Engineering and Applied Sciences, Harvard University Cambridge MA USA; e Microsoft Research AI for Science Cambridge CB1 2FB UK

## Abstract

Understanding how water wets graphene is critical for predicting and controlling its behaviour in nanofluidic, sensing, and energy applications. A key measure of wetting is the contact angle made by a liquid droplet against the surface, yet experimental measurements for graphene span a wide range, with no consensus for free-standing graphene. Here, we use a machine learning potential with *ab initio* accuracy to provide an atomistic first-principles prediction for this unsolved problem, finding a weakly hydrophilic contact angle of 72.1 ± 1.5°. More importantly, we unveil that graphene's wetting properties are highly sensitive to mechanical strain: tensile strain makes graphene significantly less hydrophilic, while compressive strain induces coherent ripples around the droplet, resulting in pronounced anisotropic wetting and contact angle hysteresis. We show that there is a strong coupling between the three-phase contact line and the intrinsic thermal ripples of free-standing graphene, which contributes to this strain sensitivity. Our results demonstrate that the wettability of 2D membranes is governed not only by their chemistry but also by their dynamic morphology, introducing a new class of wetting behaviour unique to atomically thin materials that offers an additional explanation for variability in experimental measurements. These findings reveal that mechanical strain may be a practical route to controlling wetting in 2D nanomaterials-based technologies, with promising consequences for nanofluidic and nano-filtration applications.

## Abstract 2

Mechanical strain controls how water wets graphene *via* a ripple-wetting coupling: tension makes it less hydrophilic, while compression causes the droplet to surf.

## Graphical abstract

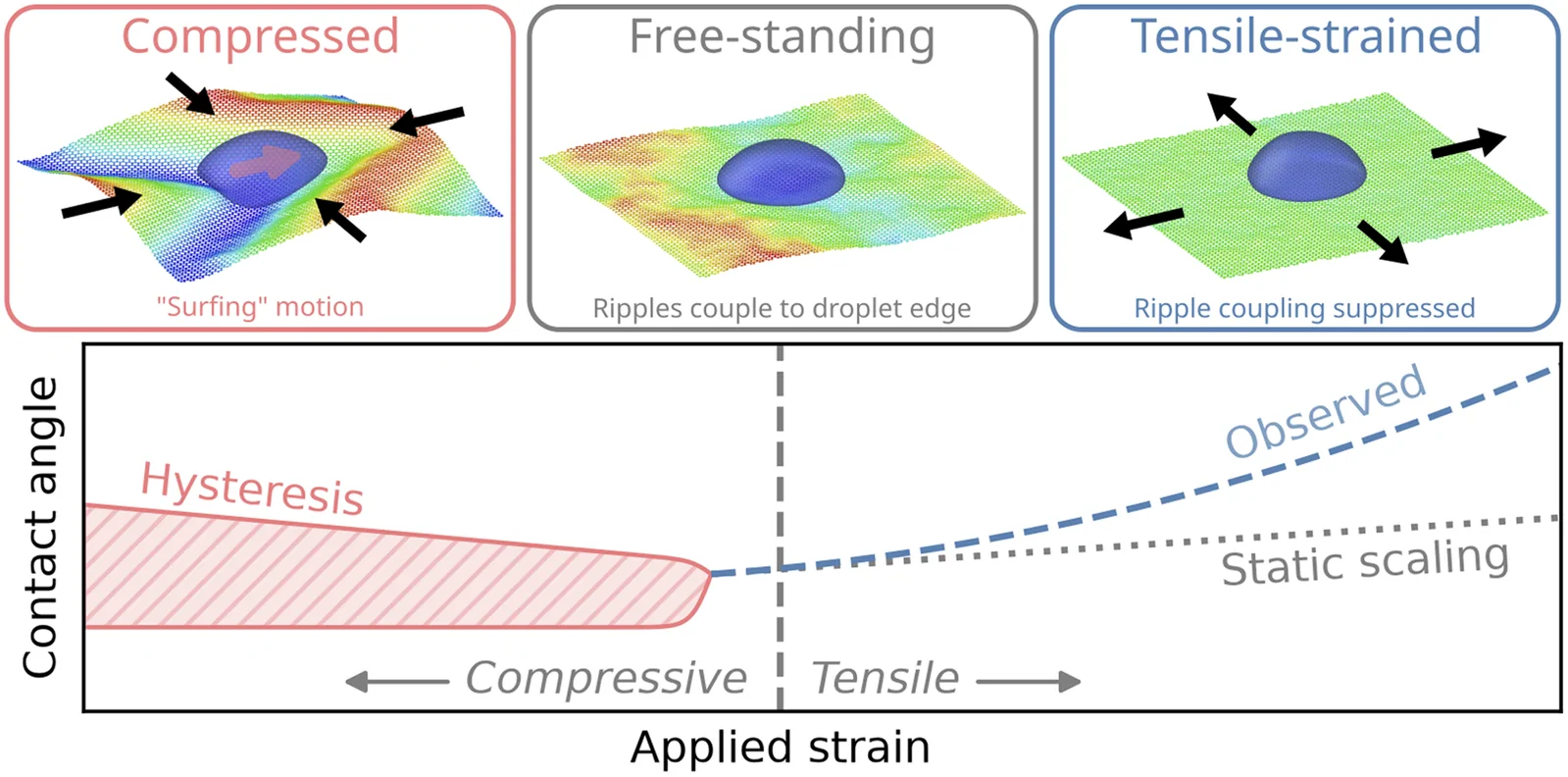

## Introduction

The water-graphene interface is one of, if not the most, scientifically fascinating and technologically important water–solid interfaces. It, for example, exhibits strongly altered dielectric properties,^1,2^ anomalous friction,^3,4^ a potential superionic state,^5,6^ and may or may not be “wetting transparent”.^7–9^ Coupled with this, it has potential applications in water filtration and desalination,^10,11^ blue energy,^12,13^ and biomolecular translocation.^14,15^ The single most important and most basic property of the interface is its wettability. Yet remarkably, after so much work, it is still not clear if graphene is hydrophobic or hydrophilic,^16,17^ nor is it known how the intrinsically rippled nature of graphene^18–20^ impacts its wettability.

The wettability is most directly quantified by the water droplet contact angle. Unfortunately, experimental measurements of the graphene-water contact angle vary drastically,^21^ from values as low as 10°^22^ to as high as 143°.^23^ Some of this variation can be attributed to the choice of substrate used to support the graphene sheet. Wetting transparency to certain materials^22,24^ might contribute, although there is disagreement as to the exact nature of this transparency.^7–9,25–27^ Some experimental studies have attempted to measure the contact angle on free-standing graphene, in order to isolate the above-mentioned effects;^28,29^ however, reported results disagree. This may be due to airborne contaminants, defects, and other imperfections during sample preparation, which have been found to affect the contact angle,^25,30–32^ providing another source of noise complicating experimental measurements.

Computational estimates of the graphene-water contact angle are likewise limited, either in system size or in accuracy. While contributing tremendously to the qualitative understanding of water-carbon behaviour, simulations based on empirical force fields yield inconsistent results for the contact angle, depending on the parametrization. For example, Taherian *et al.*^33^ report a contact angle of 101°, whereas Ma *et al.*^34^ find a contact angle of 89°, and Carlson *et al.*^21^ predict 80°. The contact angle is also sensitive to the choice of water model,^35^ further compounding these discrepancies. These issues highlight the need for simulations in which all interatomic interactions are treated from first principles.^36^ A pioneering *ab initio* molecular dynamics (AIMD) study by Li and Zeng^37^ provided early qualitative insights into this system, reporting a contact angle of 87°. While a tour de force effort for its time, true quantitative predictions were hindered by the steep computational cost of AIMD, greatly limiting the system size and thereby making finite-size effects unavoidable.^38^ More generally, the computational studies discussed above impose ‘frozen’ graphene sheets with fixed carbon atoms; yet free-standing graphene is fully flexible, and ripples thermally in order to lower its vibrational free energy.^18,19^ This flexibility is already known to impact water diffusion and transport,^39^ which is inherently linked to wetting.^40^ As such, capturing this membrane-like behaviour is essential for a realistic description of graphene's intrinsic wettability. Therefore, despite extensive experimental and computational efforts, the intrinsic graphene-water contact angle remains unresolved.

Here we provide a best-effort atomistic first-principles prediction for the contact angle of dynamical water droplets on free-standing pristine graphene using molecular dynamics (MD) simulations. By focusing on free-standing graphene, we isolate its intrinsic wetting behaviour without the influence of substrates or contamination. To this end, we develop a novel methodology to define the contact angle for a spherical nanodroplet on a non-flat surface in a spatially-resolved manner, allowing us to properly account for surface rippling in free-standing graphene. Through the use of a machine learning potential (MLP)^41–46^ trained to replicate density functional theory (DFT) calculations, we are able to simulate droplets orders of magnitude larger than in previous *ab initio* studies, which are essential for resolving and correcting the finite-size effects known to influence nanoscale contact angles.^38,47,48^

Furthermore, we study the interplay between surface ripples in free-standing graphene and the wetting of nanoscale water droplets. This interplay is probed by applying mechanical strain to the graphene sheet, thereby modifying its rippling dynamics. We discover that the application of strain has an outsized effect on the graphene-water contact angle, significantly larger than expected from energy scaling arguments. We find a two-way coupling of the dynamics of surface rippling to the three-phase contact line at the droplet edge, establishing that surface rippling plays a major role in the wetting of graphene by water. These findings imply that strain is a control lever for wettability, and contributes to the large variability in experimental measurements.

## Results and discussion

### Water contact angle on free-standing graphene is weakly hydrophilic

To determine the intrinsic wetting behaviour of water on free-standing graphene, we simulate water droplets of varying sizes and extract their contact angles (Fig. 1a). Using a MLP with first-principles accuracy at the revPBE-D3 (ref. 49 and 50) level, we are able to perform MD for systems containing up to 23k atoms and nanosecond timescales, enabling a systematic assessment of finite-size effects. These system sizes and timescales were enabled by symmetrix, a CPU-based accelerator for MLPs.^51–53^ The contact angles were determined using a novel methodology based on the geometrical intersection of the droplet's time-averaged interface with the time-averaged graphene heightmap; see Methods for details.

**Fig. 1:**
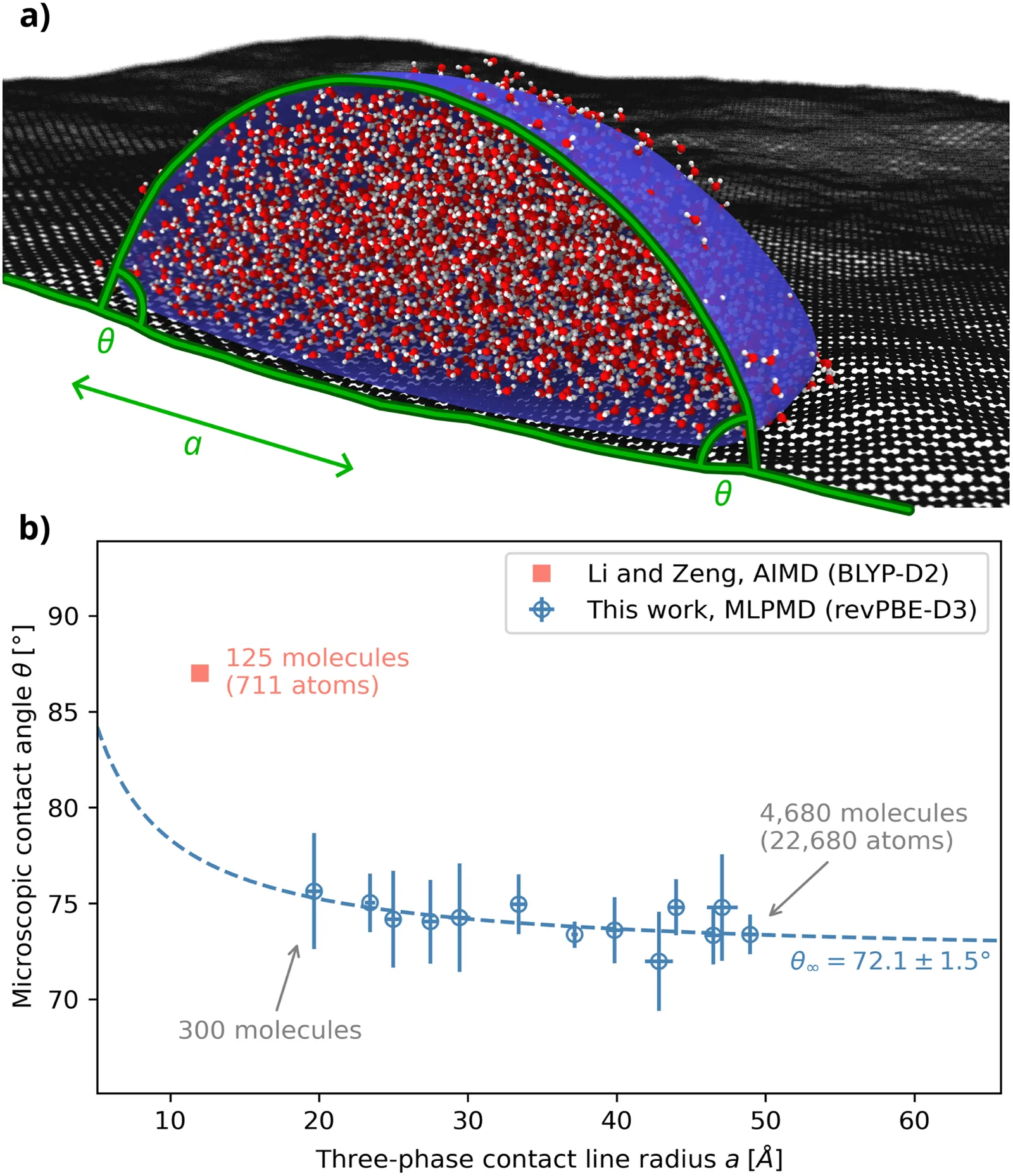
The macroscopic contact angle of water on free-standing graphene is 72.1°. (a) Cross-section of a snapshot of a spherical droplet of 4680 water molecules on a free-standing, fully-dynamical graphene sheet, with the droplet's time-averaged interface overlaid in blue. The contact angle is determined as described in the Methods section and schematically illustrated here in green. (b) The microscopic contact angle for droplets of varying size, plotted against the radius of the three-phase contact line *a*. The result obtained by Li and Zeng (ref. 37) using *ab initio* MD is also plotted for comparison. The dashed line indicates the best-fit corrections following eqn (1), whose extrapolation yields the finite-size corrected macroscopic contact angle *θ*_∞_.

For any droplet of finite size, the observed (or “microscopic”) contact angle *θ* deviates from the true macroscopic limit *θ*_∞_ due to a generalized line tension effect,^48^ which causes a linear dependence between cos *θ* and 1/*a* of the form1
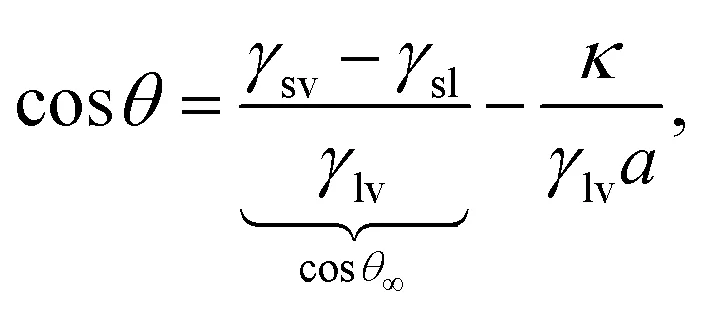
where *γ*_sv_, *γ*_sl_, and *γ*_lv_ are the surface energies of the solid-vapour, solid–liquid, and liquid-vapour interfaces respectively; *κ* is the apparent line tension;^47,48^ and *a* is the radius of the three-phase contact line.

To obtain the finite-size corrected contact angle *θ*_∞_, we therefore plot the microscopic contact angles *θ* of all simulated droplets against their corresponding radii *a* and extrapolate the resulting trend to *a* → *∞* in Fig. 1b. The apparent line tension *κ* required to make this best-fit is (7.8 ± 3.3) × 10^−12^ N, which is within the spread of previously reported line tensions in the literature.^38,54–59^ This procedure yields a macroscopic contact angle of *θ*_∞_ = 72.1 ± 1.5°. We argue that this is a good estimate for the contact angle of water on pristine, free-standing graphene, as the simulation accounts for thermal and dynamical effects, including surface ripples of the graphene sheet, at first-principles level accuracy with sufficient timescale and finite-size correction.

The contact angle directly yields the surface energy difference Δ*γ* = *γ*_sv_ − *γ*_sl_ = *γ*_lv_ cos *θ via* the Young equation.^60^ Based on the MLP's liquid-vapour surface tension (see SI), our result for the contact angle translates to a surface energy difference of 22.9 ± 1.9 mN m^−1^, where the positive value indicates graphene's weak hydrophilicity. Our estimate is consistent with the experimental measurement of van Engers *et al.*^61^ at 32 ± 8 mN m^−1^, which was obtained using a ‘direct’ pull-off force method on glass-supported epoxy-bonded graphene samples. Remaining discrepancies might be explained by the substrate effect.

We briefly compare this result with simulations using empirical force fields. For example, Ma *et al.*^34^ model the droplet using the TIP4P potential for water, plus a Lennard–Jones 6–12 potential between oxygen and carbon, fitted to a diffusion quantum Monte Carlo benchmark;^62^ they report a graphene-water contact angle of 89° based on these calculations. On the other hand, Carlson *et al.*^21^ propose a model using the SPC/E potential for water, plus a Lennard–Jones 6–12 potential also between oxygen and carbon but with different parameters, fitted to reproduce a finite-size corrected graphite-water contact angle of 60°. This predicts an 80° graphene-water contact angle, close to our estimate. As another point of comparison, Taherian *et al.*^33^ report a contact angle of 101° based on phantom-wall thermodynamic integration calculations from the Werder *et al.*^58^ potential. The disagreement between these empirical force field predictions indicates that graphene-water wetting is sensitive to the choice of parametrization, even when nominally fitted on first-principles data or experimental graphite-water contact angles. As such, our MLP-based approach provides a necessary benchmark where all degrees of freedom are described at first-principles level.

We also compare our result against the AIMD study by Li and Zeng (ref. 37), which reports a contact angle of 87° at the BLYP-D2 level of theory. However, it should be noted that their results were affected by their small system sizes, restricted to a 125-molecule droplet and a 20 ps sampling window, owing to the high cost of AIMD. Assuming a similar value of line tension for the BLYP-D2 prediction to revPBE-D3, this implies that their finite-size corrected result should be around ∼82°. Some numerical disagreement should be expected, as the underlying electronic structure of the water-graphene interaction differs, but the overall result of graphene being weakly hydrophilic is in agreement. It should also be noted that they used a spatially fixed graphene sheet, which does not account for the dynamical ripples of free-standing graphene.

In summary, we propose our macroscopic estimate of *θ*_∞_ = 72.1 ± 1.5° as a reference for the contact angle of water on pristine, free-standing graphene. Crucially, our methodology maintains a fully flexible representation of the entire system, explicitly capturing the natural thermal rippling and dynamic response of the graphene substrate. Given the known impact of surface vibrations on water diffusion and transport,^39^ this naturally raises the question of how rippling might impact the wetting behaviour.

### Strained graphene is less hydrophilic

Mechanical strain provides a natural parameter to tune the dynamical properties of free-standing graphene. For example, it is known that the application of tensile strain modifies the phonon spectrum,^63–65^ leading to a suppression of thermal ripples.^66^ Under compression, on the other hand, graphene is known to undergo a mechanical phase transition, spontaneously “buckling” and forming a long-ranged coherent ripple wave with amplitude much larger than thermal random ripples.^34,67–69^ The question, therefore, is whether these dynamical effects change the water contact angle.

We performed a series of MD simulations in which mechanical strain was applied biaxially to tune the rippling dynamics. The simulated systems explore a range of compressive and tensile strains, reaching up to 2.0% in each direction. We find that tensile strain increases the microscopic contact angle from 74.3 ± 2.8° when relaxed up to 84.8 ± 1.2° at +2.0% tensile strain (right half of Fig. 2a). The observed increase in contact angle is noteworthy, as it represents a decrease in the magnitude of the surface energy difference Δ*γ* by nearly 67% in the most extreme case. In other words, tensile strain makes graphene substantially less hydrophilic.

**Fig. 2:**
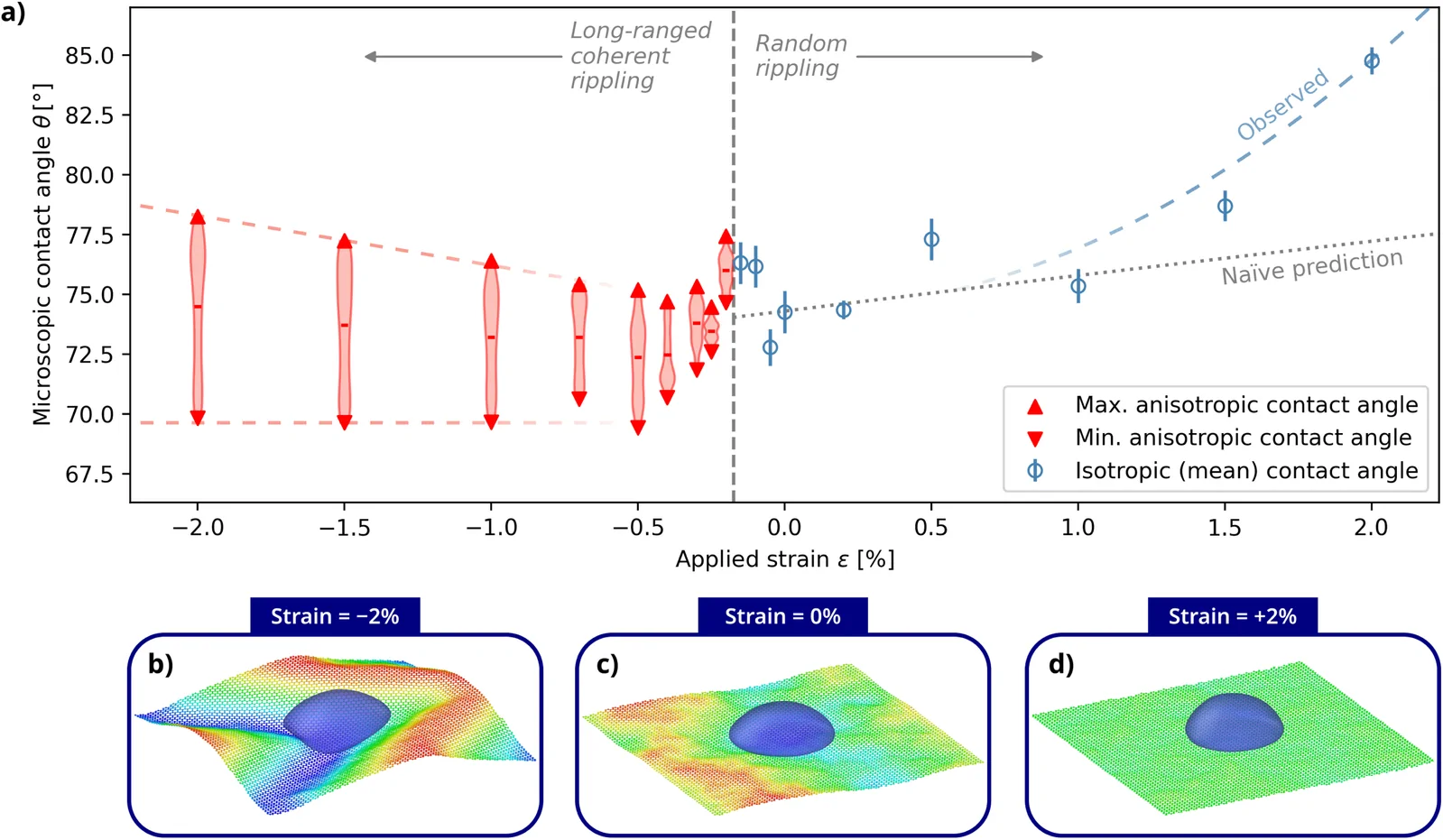
Strong strain-dependence, from compressive (negative) to tensile (positive), on the graphene-water contact angle. (a) The microscopic contact angle for a droplet of 1000 water molecules is affected by the biaxial strain applied upon the graphene sheet. When unstrained, or placed under tensile strain (right side of vertical divider), the graphene sheet ripples randomly due to thermal fluctuations, and increasing tensile strain suppresses these ripples and also increases the contact angle (dashed blue line is a guide to the eye). This increase in contact angle is significantly larger than the “naïve” prediction (dotted grey line) derived from the expected scaling of static intermolecular interaction energies with carbon density. On the other hand, compressive strains greater than or equal to approximately −0.2% (left side of vertical divider) cause the graphene sheet to form a long-ranged coherent ripple wave which the droplet “surfs”; as such the droplet exhibits continuous contact line motion, with an anisotropic distribution of contact angles in the co-moving frame, shown here as a violin plot with maximum and minimum values marked. These maximum and minimum anisotropic contact angles vary with the applied strain (dashed red lines are guides to the eye). (b–d) Representative snapshots of the graphene sheet and time-averaged droplet interface illustrated in blue under (b) −2.0% compressive strain, (c) free-standing conditions, and (d) +2.0% tensile strain, with the carbon atoms colour-coded according to their instantaneous *z*-coordinate. Snapshots (c) and (d) share the same colour scale, while snapshot (b) is shown with a different colour scale as the displacements are an order of magnitude larger. The coherent rippling of the graphene sheet, and “surfing” position of the anisotropic droplet, under compressive strain is seen in snapshot (b).

This effect cannot be explained merely by the increased interatomic spacing of the graphene sheet: naïvely, the strength of water-carbon interactions is expected to scale with the area density of carbon atoms, giving a Δ*γ* ∝ (1 + *ε*)^−2^ proportionality for strain *ε* if static effects are the only significant factor. This implies that a +2.0% strain should only drive a ∼4% decrease in Δ*γ*. Indeed, DFT calculations (see SI) show that the interaction energy of a water slab upon a fully wetted graphene sheet follows the “naïve” scaling law. Such a small change in static intermolecular interactions is therefore insufficient to explain the much larger weakening of hydrophilicity.

To further confirm whether the observed increase of contact angle under tension is related to the dynamical rippling motion of the graphene membrane, we simulated the same droplet on spatially fixed, flat graphene sheets of the same strain condition (see SI). When no strain is applied, the microscopic contact angle on the spatially fixed unstrained graphene (72.4 ± 1.5°) is within the error range of the result for dynamical free-standing graphene (74.3 ± 2.8°), which agrees with earlier findings in literature that spatially fixed and free-standing graphene have similar contact angles.^35,58^ However, this changes under tensile strain: while the contact angle on spatially fixed graphene does increase with strain, the increase is less dramatic (only to 80.8 ± 1.4°) than on dynamical graphene (84.8 ± 1.2°). This is not a fair one-to-one comparison for the dynamical component of the interfacial free energy, as spatially fixing the graphene sheet removes only the carbon degrees of freedom non-adiabatically, but this nonetheless highlights that some of the strain-driven weakening of hydrophilicity must stem from the unique dynamical properties of graphene's surface ripples and how they are modified under strain.

As for the simulations with biaxial compressive strains, we find that the graphene sheet undergoes the previously-reported mechanical phase transition for strains of approximately −0.2% or larger, where it forms a large-amplitude long-ranged coherent ripple wave (see Fig. 2b). Our observations of this phenomenon are mostly in line with previous literature,^34,67–69^ although in our case, the critical strain of this phase transition is slightly affected by the presence of the droplet. Furthermore, in this coherent rippling state, the wave travels at a roughly constant velocity plus a diffusive component, and the droplet is consequently carried along in the valley of the wave in the same “surfing” motion as first reported for force field-based simulations by Ma *et al.*^34^ This random-to-coherent rippling phase transition, and subsequent “surfing” motion, is illustrated in more detail in the SI.

Owing to this long-ranged coherent rippling of the graphene sheet, and the constant “surfing” motion of the droplet, the droplet does not relax into a static equilibrium configuration, but instead settles into a strongly anisotropic steady-state even in the long-time average. In our case, the droplet fits entirely within a single ripple trough, and its contact line motion continuously follows the surrounding crests and valleys under the constant propagation of the ripple wave. This gives rise to distinct advancing and receding sides of the three-phase contact line in the frame co-moving with the droplet's centre-of-mass, producing contact-angle hysteresis and breaking the rotational symmetry of the droplet about the *z*-axis. Consequently, the contact angle is no longer uniform, but spans a range of anisotropic values along the steady-state co-moving contact line. The distribution of these time-averaged spatially-resolved contact angles as a function of compressive strain is plotted in the left half of Fig. 2a. For sufficiently large compression, the minimum anisotropic contact angle (representing the receding boundary) is roughly constant with strain, whereas the maximum anisotropic contact angle (representing the advancing boundary) increases with strain. The mean contact angle across the three-phase contact line increases slightly with compression, as a result of this asymmetric shift.

The overall picture of Fig. 2a is that mechanical strain has a non-monotonic yet qualitatively consistent effect: both tensile and compressive strain increase the contact angle, meaning that graphene becomes effectively less hydrophilic under either type of strain. It should be noted that, for compressive strains, the dynamic angles at the receding and advancing boundaries cannot be directly compared to the static equilibrium angles under tensile strains, as contact angle hysteresis is driven by the kinetics of contact line motion rather than the equilibrium interfacial thermodynamics.^70^ Nonetheless, the two-sided trend of Fig. 2a shows that mechanical strain has an outsized effect on graphene's wettability. Given the strong modulation of surface dynamics by strain, we turn next to a detailed analysis of the coupling of surface rippling and water droplet formation.

### Two-way coupling of surface ripples to the droplet edge

In all simulations for either unstrained or tensile strained graphene, the presence of the water droplet induces a persistent distortion on the graphene sheet, resembling a “footprint” (see Fig. 3b). The distortion is reduced, but never eliminated, by applying tensile strain (see Fig. 3c). This latent curvature of the graphene surface towards the droplet can be attributed to weakly hydrophilic attractions, which favour an increased graphene-water contact area compared to a perfectly flat surface. Such curvature must be localized in the vicinity of the droplet edge in the limit of large droplet sizes, due to translational symmetry along the *xy* plane in both the deep droplet interior and the faraway unwetted region forbidding any curvature.

**Fig. 3:**
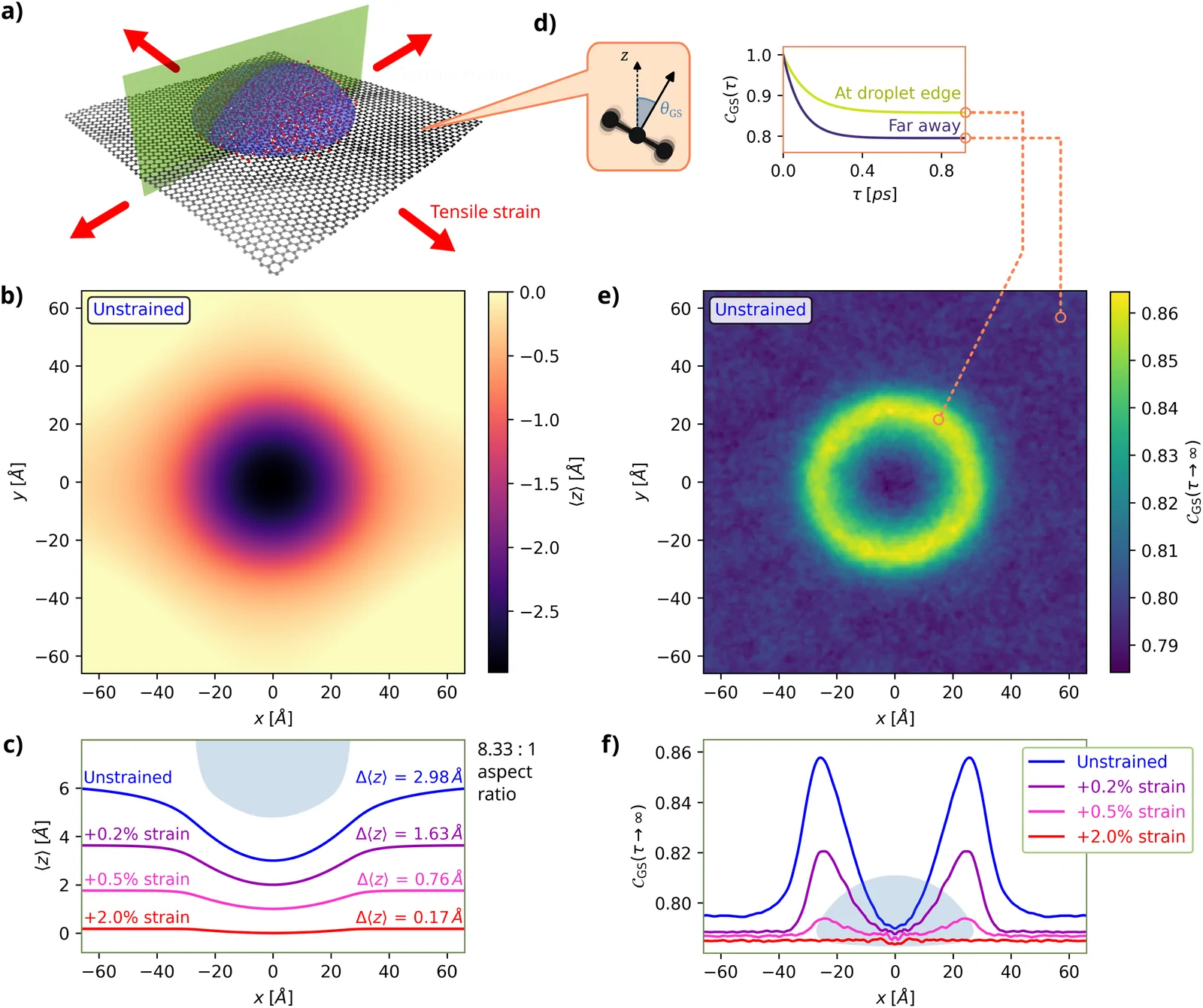
The “footprint” of the water droplet on graphene and its effects on graphene's rippling dynamics under strain. (a) A representative snapshot of a droplet of 1000 molecules, with tensile strain applied to the graphene sheet. The translucent green plane indicates the cross-section that panels (c) and (f) pertain to. (b) The time-averaged graphene heightmap (TAGH), representing the average *z*-coordinate of the carbon atoms in droplet-centred coordinates, for the unstrained case. (c) The TAGH along the cross-section for each strain condition, with the time-averaged droplet interface for the unstrained case shown at the top. The graph is plotted with an 8.33 : 1 aspect ratio to highlight the distortion of the sheet. (d) The dynamics of surface rippling were studied *via* the graphene sheet's local inclination angle *θ*_GS_ relative to the *z*-axis, and its normalized temporal autocorrelation function 
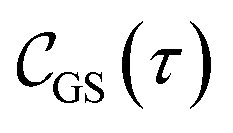
 as defined in eqn (5); this is further elaborated in Methods. The graph shows two examples of 
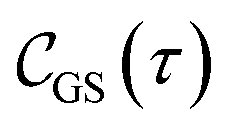
 plotted against *τ* for two locations on the free-standing graphene sheet, which decay exponentially from unity to some long-time limit. (e) The long-time limit 
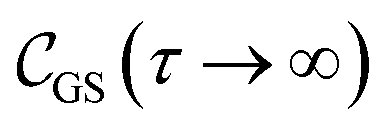
 of the graph from panel (d), illustrated for all locations on the graphene sheet for the unstrained case. This “long-time inclination correlation” peaks at the droplet edge and returns to a uniform background value everywhere else, including at the droplet centre. (f) The long-time inclination correlation 
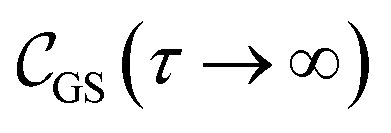
 along the cross-section for each strain condition. The time-averaged droplet interface for the free-standing case is overlaid in the background, to illustrate the location of the droplet edge.

The graphene-water-vacuum interface along the droplet perimeter, *i.e.* the three-phase contact line, must also perturb the dynamics of graphene's thermal ripples. This arises from the breaking of translational symmetry at the contact line, disrupting Bloch symmetry for all phonon dispersions.^71^ To study this, we examine the long-time inclination correlation of the graphene sheet, denoted 
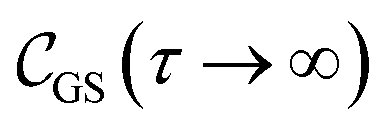
, which was first introduced by Thiemann *et al.*^72^ as a spatially-localized indicator of phase transitions in rippling dynamics; see Methods for details. The long-time inclination correlation quantifies how strongly the sheet's ripples are correlated over time, with values close to 1 suggesting a constrained local inclination and static rippling, and lower values suggesting greater flexibility and dynamic rippling. For reference, free-standing unwetted graphene at 300 K has a uniform value of 
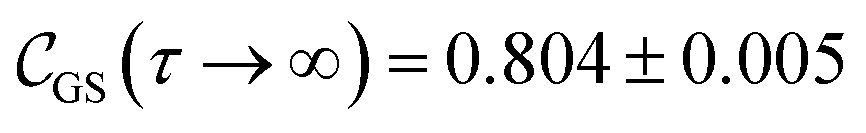
, and a graphene sheet fully covered by water has 
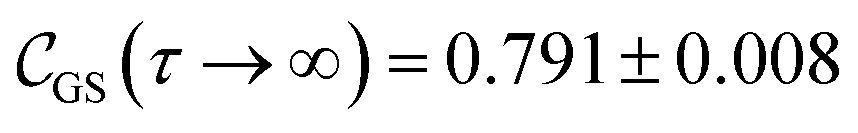
. In the presence of a droplet, however, 
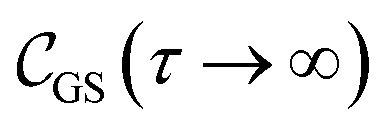
 exhibits a pronounced, highly localized peak of approximately ∼0.86 at the droplet perimeter (Fig. 3e). This enhancement suggests that the wetting-induced curvature leads to a local stiffening of the membrane, ‘freezing out’ orientational fluctuations of ripples in the direction tangential to the contact line.

Under the application of tensile strain, we observe a decrease in the magnitude of thermal random ripples, as consistent with literature.^66^ Moreover, strain leads to a flattening of the droplet-induced curvature, seen in Fig. 3c. Finally and most strikingly, strain suppresses the localized perturbation of 
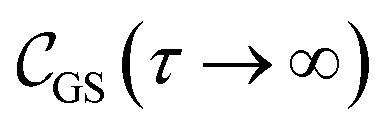
, seen in Fig. 3f. In the most extreme case of the +2.0% tensile strain, the time-averaged *z*-coordinate of the graphene sheet only has a displacement of 0.17 Å compared to 2.98 Å in the unstrained case, and there is no perturbation seen in 
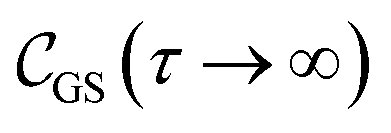
 at all. A similar trend is seen under compressive strains, where the localized perturbation of 
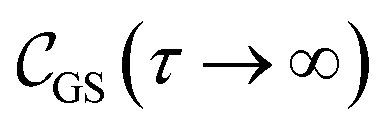
 at the droplet perimeter vanishes with increasing compression, though in this regime the long-ranged coherent ripple wave presents a non-uniform “background” for the long-time inclination correlation (see SI).

Although some disturbance of rippling dynamics at the droplet boundary is expected from symmetry arguments alone, the magnitude and sharpness of the observed jump in 
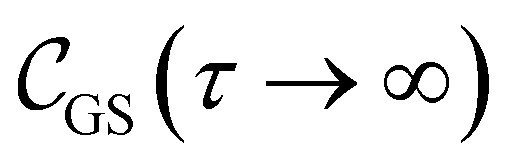
 for the unstrained case is striking. Rather than transitioning smoothly from the fully wetted value of ∼0.79 to the unwetted value ∼0.80, the correlation increases dramatically only at the droplet edge. Furthermore, this disturbance is sensitive to modifications of the graphene sheet's vibrational spectrum *via* mechanical strain. This behaviour points toward a two-way coupling in which wetting modifies graphene's ripples, and the ripples, in turn, contribute to the free-energy balance at the contact line. We argue that this mechanism explains the anomalous increase of contact angle under tensile strain, as shown in Fig. 2a on dynamical graphene, beyond any effect attributable to spatially fixed graphene.

The coupling of water wetting to surface ripples is reminiscent of the “dripplons” reported by Yoshida *et al.*^73^ A dripplon is a localized water droplet, formed within graphene-nanoconfined water with an accompanying deformation of the graphene sheets; they are created by water's layering preference being stronger than the graphene bending energy. In particular, dripplons represent a coupling between confined water's mixed hydration states and graphene's flexibility, which is a mechanism unique to flexible confining materials, as opposed to rigid materials which prefer interstratification of the mixed hydration states. In our case, the unconfined droplets result in coupling between the three-phase contact line and graphene's surface ripples instead, being uniquely enabled by the flexibility of graphene.

## Conclusion

In this work, we used a MLP with first-principles accuracy to perform nanosecond-scale MD simulations of water droplets on graphene to deliver a best-effort prediction of the contact angle of 72.1 ± 1.5°. These results show that free-standing graphene is weakly hydrophilic, at the revPBE-D3 level of theory. We expect this contact angle estimate to be a reference for future experiments, in particular for settling the “wetting transparency” controversy by measuring the contact angle on free-standing graphene unsupported by any substrate. While the exact value of the contact angle may potentially depend on the description of the underlying electronic structure, we expect the general conclusions to be robust.

Furthermore, we show that the application of mechanical strain has a strong effect on the wetting of dynamical graphene. Tensile strain causes the water contact angle for a nanoscopic droplet of fixed size to increase drastically, implying that tension makes graphene less hydrophilic. This increase of contact angle is only partially accounted for by the weakening of static water-graphene interactions, and is also related to dynamical rippling of the graphene sheet. We demonstrate that the presence of a water droplet not only induces an average curvature in the membrane, but also causes a localized disturbance of the vibrational dynamics of the graphene sheet in the vicinity of the droplet boundary, thus revealing a two-way coupling between wetting at the three-phase contact line and graphene surface ripples. Tensile strain then results in a suppression of this dynamical perturbation, which contributes to the strong increase of contact angle.

The application of compressive strain induces a phase transition from thermally random ripples to long-ranged coherent rippling. For droplets of lengthscale comparable to the wavelength, the coherent wave ‘straddles’ the droplet, causing the droplet to “surf” the valley of the wave. This wave-driven motion gives rise to an anisotropic droplet geometry with a range of non-uniform contact angles, and in particular the advancing contact angle increases with compression due to corrugation stiffening. For wetting at lengthscales much larger than the wavelength of the coherent wave, we imagine that the nanoscopic roughness of the compressed sheet might also lead macroscopically to either the Wenzel or Cassie–Baxter wetting states,^74–76^ although this only occurs far beyond the nano scale.

Our results show that mechanical strain affects the contact angle non-monotonically, and is an important parameter in studying the wetting of graphene. Qualitatively similar results, where solid–solid interfacial energies can be affected by strain, have been previously demonstrated in the literature;^77–79^ these reported results, however, are driven by mechanical effects limited to solid–solid interfaces, *e.g.* lattice mismatch and surface stress. Such mechanisms do not apply to the graphene-water system, which is laterally isotropic and atomically thin. Instead, we propose that mechanical strain has a significant effect on wetting *via* its coupling to graphene surface ripples (ZA phonons), even though such modes are typically neglected in continuum wetting theories. This may be a further explanation for the large range of reported experimental results, where the contact angle seems to be greatly affected by the substrate hosting the graphene. While strain can be controlled experimentally to fairly good precision,^80^ some degree of strain is always unavoidable in real experiments. As such, even if graphene is fully chemically opaque to wetting, the presence of the substrate (and any strain induced by lattice mismatch or thermal expansion) fundamentally alters the vibrational spectrum on the graphene and thus changes its wetting properties.

These observations suggest that, in general, wetting on a 2D membrane is fundamentally different compared to rigid solids: wetting is governed not only by surface chemistry — where *e.g.* the presence of chemically polar interactions is already known to greatly affect hydrophobicity and hydrophilicity^81^ — but also by vibrational entropy. This introduces a new class of wetting behaviour unique to atomically thin materials. On the flip side, our results also suggest that it might be possible to modulate and control the wetting of 2D nanomaterials, particularly graphene, *via* mechanical strain engineering for potential technological applications. We propose that such “strain-dependent hydrophilicity” could be used to develop nanofluidic pumps for transporting water through graphene-confined or carbon nanotube-confined systems.^4,82^ This could be confirmed experimentally using *e.g.* high-resolution atomic force microscopy (AFM) to detect the wetting-induced curvature of a nanodroplet,^83,84^ or electron spectroscopy to detect ripple suppression at the droplet edge.^85,86^

## Methods

### Machine learning potential

Throughout this work, we use the same MLP model as ref. 87. This MLP was developed using the MACE architecture^88–90^ with 128 invariant channels (*L* = 0), two layers, and a 6 Å cutoff distance per layer. The model thus captures semi-local interactions through an effective receptive field of 12 Å.

To accurately represent the potential energy surface, the MLP was trained using energies and atomic forces obtained from DFT calculations using the CP2K/Quickstep code.^91^ We specifically used the revPBE functional^49^ with D3 dispersion correction with zero damping,^50^ due to its robust performance in reproducing the structure and dynamics of liquid water,^92–94^ while also effectively capturing protonic defects and the interaction energies between water and graphene.^95^

Training data included a wide variety of configurations representing different carbon-water environments, such as: bulk and implicitly confined water; free-standing graphene sheets, and AA and AB stacked graphene sheets of varying distance; water on graphene; and water confined between graphene sheets or within carbon nanotubes. In total, the training set comprised 5845 structures, yielding root-mean-square errors of 0.9 meV per atom for energies and 26.3 meV Å^−1^ for forces.

The MLP was extensively benchmarked against unseen test data, and shown to reproduce an accurate description of water across a wide range of temperatures, demonstrating its transferability and accuracy for the thermodynamic landscape of bulk liquid water. It was also shown to be able to predict the bending rigidity of graphene, which is critical for studying graphene rippling dynamics.

Further details of this MLP development and benchmarking are provided in the SI.

### Molecular dynamics simulations

All MD simulations reported herein with the MLP were performed using the LAMMPS^96^ software patched with symmetrix^51–53^ — an optimized C++ and Kokkos implementation that accelerates machine learning potentials for efficient large-scaled inference — for fast CPU evaluation on the ARCHER2 (ref. 97) cluster. Unless explicitly stated otherwise, all simulations were performed in the NVT ensemble at a temperature of 300 K. A time step of 1 fs was employed, and simulations utilized a Nosé–Hoover thermostat with damping time 100 fs. The total simulation time, across all configurations, was 48.88 ns.

The simulations reported for the finite-size corrected water contact angle on free-standing graphene consist of 13 different spherical water droplets, of sizes ranging from 300 to 4680 water molecules, placed on a free-standing graphene sheet of dimensions 154.0 Å × 148.2 Å; the total number of atoms thus range from 9540 to 22 680. Each configuration was simulated for 1.30 ns, with the first 0.10 ns used for equilibration and the remaining 1.20 ns broken up into 5 statistically-independent blocks for the contact angle analysis and uncertainty estimate.

The simulations reported for the effects of mechanical strain on the contact angle consist of the droplet of 1000 water molecules placed on the same graphene sheet, but with mechanical strain applied biaxially by horizontally scaling the *x* and *y* directions of the fixed periodic boundaries. Each configuration thus contains 11 640 atoms. Each configuration was simulated for 1.06 ns, with the first 0.10 ns used for equilibration and the remaining 0.96 ns broken up into 4 statistically-independent blocks for the contact angle analysis and uncertainty estimate.

Further details of the MD simulations are provided in the SI.

### Obtaining the contact angle on non-flat surfaces

In previous works, the contact angle is typically defined atomistically using the angle of intersection between the Gibbs equimolar dividing surface of the liquid-vapour interface, and the solid surface.^21^ Various methods exist for calculating this Gibbs dividing surface, with the most common approaches relying on slicing and binning molecular positions relative to the (fixed, flat) solid surface, and then fitting sigmoidal distributions to the binned densities, before extracting the angle of intersection from a best-fit sphere of the Gibbs dividing surface.^58^

These approaches, however, require a planar solid surface and thus cannot be applied to free-standing graphene. Instead, we introduce a novel method inspired by the Willard–Chandler instantaneous interface^98^ to compute an effective dividing surface in a symmetry-agnostic manner.

We consider only the oxygen atoms to describe the positions of the liquid water molecules. In this section, we work in droplet-centred coordinates, *i.e.* the coordinate system is translated such that, at all times *t*, the centre-of-mass of the droplet is located along the *x* = *y* = 0 axis.

Given the positions **R**_*i*_(*t*) of the *i*^th^ oxygen atom at time *t*, the coarse-grained density field at a space-time point **r**, *t* is defined as2
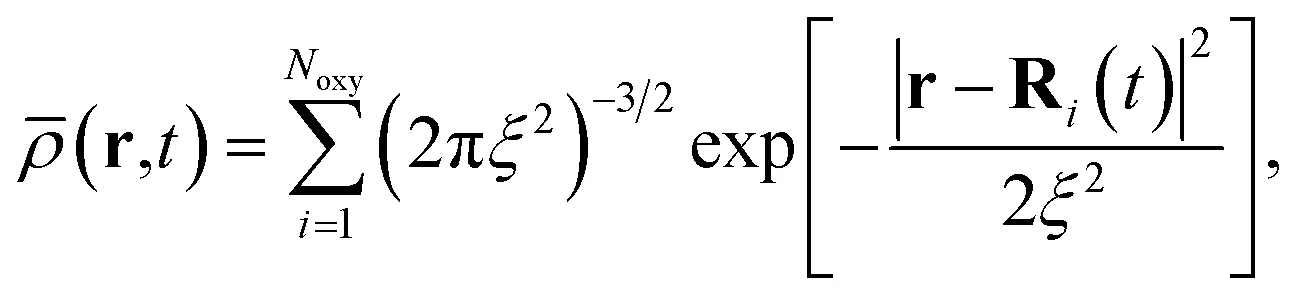
where *ξ* is the coarse-graining length. The Willard–Chandler instantaneous interface is then defined as the 2-dimensional isosurface **r** = **s** where the coarse-grained density field reaches a cut-off value *ρ̄*(**s**, *t*) = *c*; typically *c* is chosen to be half of the bulk liquid density. The values of the parameters are *ξ* = 2.4 Å and *c* = 0.016 Å^−3^ for water.^98^

Here we introduce the notion of the time-averaged interface to similarly be the isosurface of the time average of the density field, that is:3
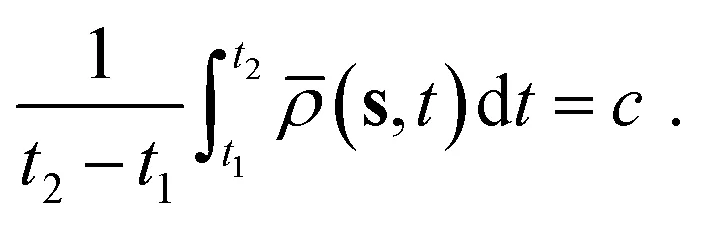


For sufficiently long times, the time-averaged interface converges almost to the Gibbs dividing surface except “rounded” by lengthscale *ξ*. To calculate the interface, instead of interpolating eqn (2) over a spatial grid as conventionally done for the Willard–Chandler method,^98^ we use a ray-tracing binary search algorithm (detailed in the SI) to sample points on the interface in logarithmic runtime. This approach is therefore advantageous over the Gibbs dividing surface, in that no initial assumptions are made on the geometry of the droplet other than being simply connected.

Next, we define the instantaneous graphene heightmap to be a piecewise cubic function *h*_GS_(*x*, *y*; *t*) satisfying the following conditions:

• *z*_*i*_(*t*) = *h*_GS_(*x*_*i*_(*t*), *y*_*i*_(*t*); *t*) for all carbon atoms at position **R**_*i*_(*t*) = (*x*_*i*_(*t*), *y*_*i*_(*t*), *z*_*i*_(*t*)) at time *t*;

• *h*_GS_(*x*, *y*; *t*) is *C*^1^ smooth over all *x* and *y* for all *t*;

• Amongst all functions which satisfy the above two, *h*_GS_ is the one which minimizes total curvature 
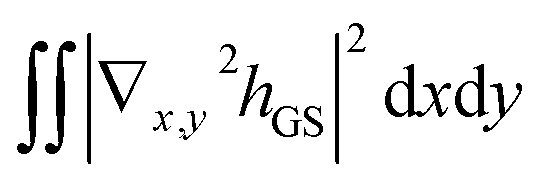
;

This is achieved implicitly using a Clough–Tocher interpolation scheme.^99^ The time average of this instantaneous heightmap is then taken, yielding 〈*h*_GS_(*x*, *y*)〉_*t*_ as the time-averaged graphene heightmap or TAGH for short.

With these definitions, the contact angle is obtained by scanning for the time-averaged interface eqn (3) along randomly-selected rays which do not intercept the TAGH, fitting a least-squares best-fit sphere to these sampled points, and calculating the intersection angle between this best-fit sphere and the TAGH. This procedure can either be performed isotropically, in which case the TAGH is averaged over azimuthal rotations to produce a radially-symmetric function 〈*h*_GS_(*r*)〉_*t*_ with which the intersection is computed; or anisotropically, to produce a range of contact angles along the entire span of the intersection line.

### Graphene sheet long-time inclination correlation

The graphene sheet long-time inclination correlation, first introduced by Thiemann *et al.*,^72^ is a spatially-localized measure of the degree to which graphene surface wave fluctuations are constrained. Specifically, it is the long time limit of the temporal autocorrelation function of the local inclination angle *θ*_GS_(*x*, *y*; *t*), *i.e.* the angle between the normal vector of a regularized instantaneous graphene heightmap and the *z*-axis

4 *θ*_GS_(*x*, *y*; *t*) = arctan(|∇_*x*, *y*_*h̄*_GS_(*x*, *y*; *t*)|),

where the regularized heightmap *h̄*_GS_ = *h*_GS_ ∗ *Π*_σ_ is the convolution of the instantaneous graphene heightmap with a uniform disk function *Π*_σ_ of radius *σ*, which has the effect of smoothing out sharp gradients. We choose *σ* = 4.5 Å to match the procedure employed by Thiemann *et al.*^72^

The normalized temporal autocorrelation is thus5
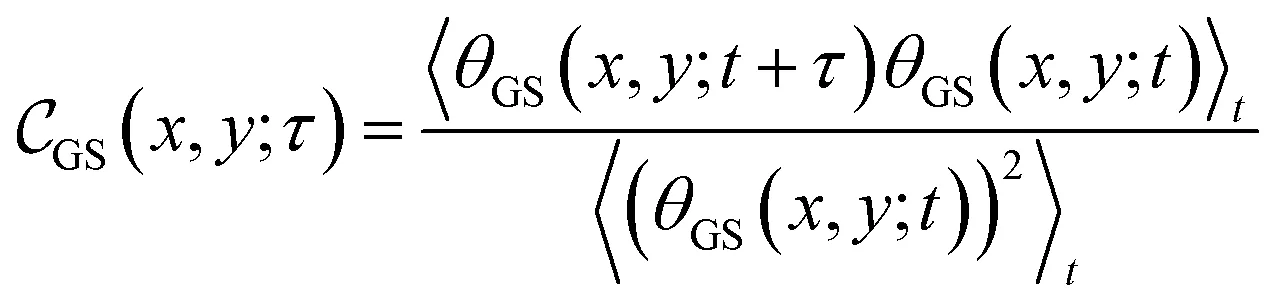
and the long-time inclination correlation is the limit *τ* → *∞* as obtained by fitting exponential decay curves to the temporal autocorrelation.

## Author contributions

D. W. L., A. M., F. L. T., and C. S. conceived of the presented ideas. D. W. L. carried out all simulations, created the contact angle methodology, and performed the data analysis. X. R. A. performed the reference DFT calculations for the MLP training data, and also developed and benchmarked the MLP. X. R. A. also assisted D. W. L. with the DFT calculations for single molecule interaction energies (in SI). W. C. W. created the symmetrix implementation. A. M. and F. L. T. contributed to the interpretation of the results. C. S. supervised the project. D. W. L. took the lead in writing the manuscript. All authors provided critical feedback, and helped shape the research, analysis, and manuscript.

## Conflicts of interest

The authors declare no competing interests.

## Supplementary Material

SC-OLF-D6SC05377G-s001

## Data Availability

All data required to reproduce the findings of this work is available on GitHub: https://github.com/fast-group-cam/data_graphene_water_contact_angle. Supplementary information (SI): details of molecular dynamics simulations, machine learning potential development & validation, properties of liquid water predicted by the MLP model, code implementation for obtaining the contact angle on non-flat surfaces, comparison of contact angle methodologies on unstrained spatially-fixed flat graphene and unstrained dynamical graphene, interaction energy of water with graphene under tensile strain, contact angle on spatially-fixed graphene *versus* dynamic graphene under tensile strain, “surfing” motion of droplet on long-ranged coherent ripple waves, droplet footprint on compressed graphene. See DOI: https://doi.org/10.1039/d6sc05377g.
