# Supplementary material for: Strain-dependent wetting of graphene
Source: Chem Sci. 2026 Oct 20. Online ahead of print. doi: 10.1039/d6sc05377g (PMC13716132; doi:10.1039/d6sc05377g)
Supplement: Supplementary file 1 [file SC-OLF-D6SC05377G-s001.pdf]

Supplementary Information (SI) for Chemical Science.
This journal is © The Royal Society of Chemistry

Supporting Information: Strain-Dependent Wetting of Graphene

Darren Wayne Lim,[†,‡] Xavier R. Advincula,[¶,†,‡] William C. Witt,[§] Angelos Michaelides,[¶,‡] Fabian L. Thiemann,[‖,†] and Christoph Schran[*,†,‡]

†*Cavendish Laboratory, Department of Physics, University of Cambridge, Cambridge, CB3 0HE, UK*

‡*Lennard-Jones Centre, University of Cambridge, Trinity Ln, Cambridge, CB2 1TN, UK*

¶*Yusuf Hamied Department of Chemistry, University of Cambridge, Lensfield Road, Cambridge CB2 1EW, UK*

§*Harvard John A. Paulson School of Engineering and Applied Sciences, Harvard University, Cambridge, MA, USA*

‖*Microsoft Research AI for Science, Cambridge, CB1 2FB, UK*

E-mail: cs2121@cam.ac.uk

Contents

Details of molecular dynamics simulations **S4**

Droplets of varying sizes, no strain applied S4

Droplets of 1,000 water molecules, varying strain applied S7

Graphene, with and without water S7

Bulk water, and water slabs S8

Machine learning potential **S13**

Model development S13

Model validation S14

Properties of liquid water predicted by the MLP model **S19**

Density and radial distribution function S19

Surface tension S21

Code implementation for obtaining the contact angle on non-flat surfaces **S23**

Algorithm for determining the time-averaged interface S23

Best-fit sphere of the faraway time-averaged interface S25

Finding the graphene heightmap S27

Calculating the anisotropic contact angle S28

Calculating the isotropic contact angle S29

Parameter sensitivity check S29

Comparison of contact angle methodologies on spatially-fixed flat graphene and dynamical graphene (unstrained) **S32**

Interaction energy of water with graphene under tensile strain **S34**

Contact angle on spatially-fixed graphene versus dynamical graphene under +2.0% tensile strain **S38**

"Surfing" motion of droplet on long-ranged coherent ripple waves **S40**

Droplet footprint on compressed graphene **S43**

References **S45**

Details of molecular dynamics simulations

Except where otherwise noted, all MD simulations performed in this work were based on the MLP, and performed using the LAMMPS[S1] software patched with the `symmetrix`[S2] library for fast, scalable, and highly parallelisable CPU evaluation of the MACE MLP. This allows us to perform MLP-based MD at a very large scale, thus accessing the approaching *ab initio* accuracy of the MLP's potential energy surface without needing to expend unreasonable computational effort.

All simulations used orthorhombic cells with periodic boundary conditions in all three dimensions. A timestep of 1 fs was employed throughout. Most simulations were performed in the NVT ensemble at a temperature of 300 K, which was maintained by a Nosé–Hoover thermostat with damping time 100 fs as implemented in LAMMPS; one simulation was performed in the NpT ensemble at a pressure of 1 atm, which was maintained by a Nosé–Hoover barostat with damping time 1.000 fs.

Droplets of varying sizes, no strain applied

The graphene sheets were constructed by repeating the unit cell dimensions $a = 3d_c$ and $b = \sqrt{3}d_c$ along the x and y directions respectively, where $d_c = 1.426$ Å is the carbon-carbon bond length obtained by energy minimization of the MLP at 0 K (rather than the experimentally measured value of $d_c = 1.42$ Å[S3]). As such, the 154.0 Å × 148.2 Å graphene sheets were obtained by repeating the unit cell 36 times in the x direction, and 60 times in the y direction. This yields a graphene sheet containing 8,640 carbon atoms. Sufficient vacuum space was added in the z direction to ensure that periodic images across the z direction do not interact.

The spherical water droplets of varying sizes were simulated in a sequential approach, where the equilibration of each droplet was used as the starting point for the next smaller droplet. The first system to be created was the 4,680 water molecules droplet, which was

set up by generating a 77.0 Å × 74.1 Å × 24.6 Å box of liquid water on top of the graphene sheet, and then equilibrating for 200 ps. This equilibrated droplet was thus the starting point for the production run of 1.20 ns for the measurement of the contact angle. At the same time, the 4,300 water molecules droplet was generated by deleting 380 water molecules at random from the 4,680 water molecules droplet, and then further equilibrated for 100 ps before starting the 1.20 ns production run.

Smaller droplets were similarly generated by deleting the relevant number of water molecules from the equilibrated configuration of the next bigger droplet, and then equilibrating for 100 ps more. A total of 13 different droplet sizes were investigated. All of the simulations described above were performed using the MLP with fully flexible, dynamical free-standing graphene. This set of simulations are relevant to obtaining the finite-size corrected contact angle for water on pristine, free-standing graphene.

Further to this, in order to investigate if the contact angle is significantly affected on free-standing graphene as opposed to spatially fixed, flat graphene, three more droplets (of sizes 4,680; 2,000; and 1,000 water molecules) were prepared on spatially fixed graphene sheets. These droplets were prepared by taking the corresponding starting state of the free-standing graphene simulation, resetting the positions of the carbon atoms back to the $z = 0$ plane, and translating the water molecules along the z-axis so as to put the closest water molecule 1 Å away from the graphene sheet. The droplets were then equilibrated for 100 ps before starting the 1.20 ns production run, with the graphene held fixed always. This set of simulations are relevant for comparing the contact angles between free-standing and spatially fixed graphene sheets, and also for comparing the contact angle methodology introduced in this work to an established literature method.

A flowchart summarizing this sequence of simulations is presented in Figure S1; the configurations are listed in Table S1.

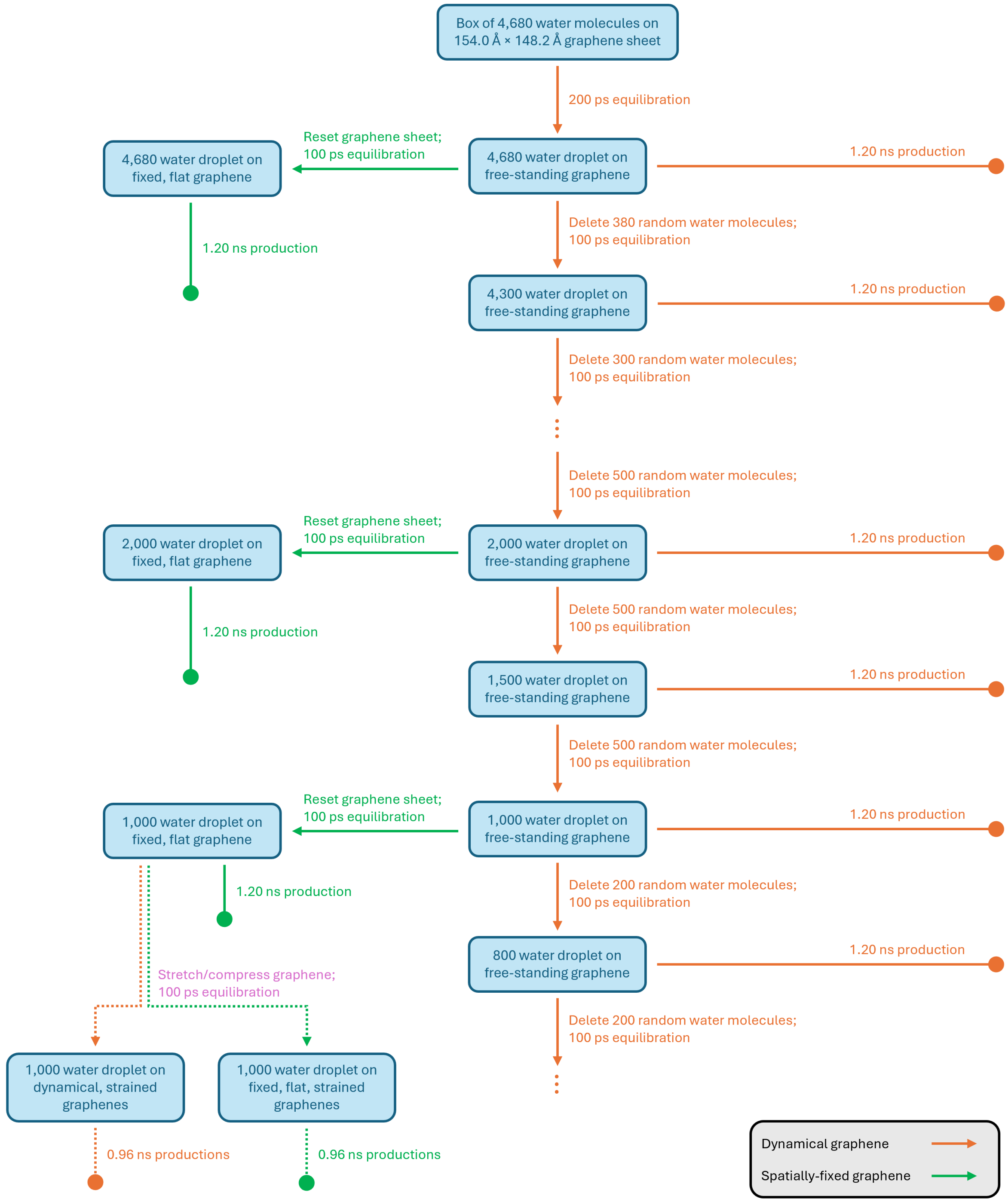

Figure S1: **Flowchart for the sequence of simulations performed in this work involving water droplets of varying size on strained and unstrained graphene sheets.** Arrows are colour-coded based on whether the graphene sheet was simulated dynamically or kept fixed in place.

Droplets of 1,000 water molecules, varying strain applied

To study how biaxial mechanical strain on the graphene sheet affects the wetting of graphene, we simulated an applied strain by re-scaling both the simulation periodic unit cell, and also the initial positions of the carbon atoms, in the x and y directions but not in the z direction. The fixed periodic boundaries thus maintain a constant-strain condition.

These strained simulations were prepared by taking the equilibrated droplet of 1,000 water molecules on spatially fixed, flat, unstrained graphene (equilibrated using the MLP), performing the relevant re-scaling in the x and y directions, and then propagating the MD simulation with fully dynamical graphene. Values of tensile strains applied are: $\varepsilon = +0.20\%$, $+0.50\%$, $+1.00\%$, $+1.50\%$, $+2.00\%$. Values of compressive strains applied are: $\varepsilon = -0.10\%$, -0.15%, -0.20%, -0.25%, -0.30%, -0.40%, -0.50%, -0.70%, -1.00%, -1.50%, -2.00%. The strained systems were equilibrated for a further 100 ps, before starting the 0.96 ns production run. This set of simulations are relevant for elucidating the coupling between graphene surface rippling and the three-phase contact line, and the effect of mechanical strain on the droplet contact angle.

An additional "strained + spatially fixed" simulation was also prepared, at the tensile strain of $\varepsilon = +2.0\%$, using the same procedure above but propagating the MD simulation with the graphene sheet still spatially fixed. This simulation is relevant for comparing the effect of strain on the droplet contact angle with or without the dynamical motion of the graphene sheet.

Graphene, with and without water

To measure the long-time inclination correlation $\mathcal{C}_{\mathrm{GS}}(\tau \to \infty)$ associated with pristine, free-standing graphene both unwetted and wetted uniformly, we also ran short simulations of (a) the graphene sheet on its own, and (b) covered uniformly on one side with a uniform water slab. In case (a), the graphene sheet was constructed the same way as in the water droplet simulations, while in case (b), a smaller 38.5 Å × 37.1 Å graphene sheet of 540 carbon atoms

was used in order to save on computational cost. The graphene sheet was then covered uniformly by a box of 1,170 water molecules. In both cases, the system was equilibrated for 100 ps, before starting the 300 ps production run.

For the dry free-standing graphene sheet, the mean value of the long-time inclination correlation $\mathcal{C}_{\mathrm{GS}}(\tau \to \infty)$ across the sheet was 0.804 ± 0.005. For the uniformly-wetted graphene sheet, the water slab was seen to have an average thickness of $\sim$25 Å, and the mean value of $\mathcal{C}_{\mathrm{GS}}(\tau \to \infty)$ was 0.791 ± 0.008.

Bulk water, and water slabs

To measure the properties of bulk liquid water as predicted by the MLP, we:

- simulated a box of 64 water molecules with fully periodic boundaries, to find the radial distribution functions of bulk liquid water under NVT conditions;
- simulated a box of 520 water molecules with fully periodic boundaries, to find the radial distribution functions and equilibrium density of bulk liquid water under NpT conditions;
- simulated five slabs of liquid water, of varying sizes, to find the surface tension.

Table S1: **Detailed overview of the simulations presented in this work.** For each system, we report, where relevant, the total number of atoms N_{atoms}; the corresponding number of water molecules N_{H_2O}; the equilibration time t_{eq}; the simulation production time t_{sim}; and the pressure p. All systems used the same timestep $\delta t = 1\,\text{fs}$ and same temperature $T = 300\,\text{K}$.

**System** **(Dimensions)**	**Simulation details**	**Illustration of largest example**
Droplets on free-standing graphene (154.0 Å × 148.2 Å ×151.1 Å)	$N_{\text{atoms}} \in \{22680; 21540; 20640; 19140; 17640; 16140; 14640; 13140; 11640; 11040; 10440; 10140; 9540\}$ $N_{H_2O} \in \{4680; 4300; 4000; 3500; 3000; 2500; 2000; 1500; 1000; 800; 600; 500; 300\}$ $t_{\text{eq}} = 100\,\text{ps}$ (+100 ps for largest droplet) $t_{\text{sim}} = 1.20\,\text{ns}$	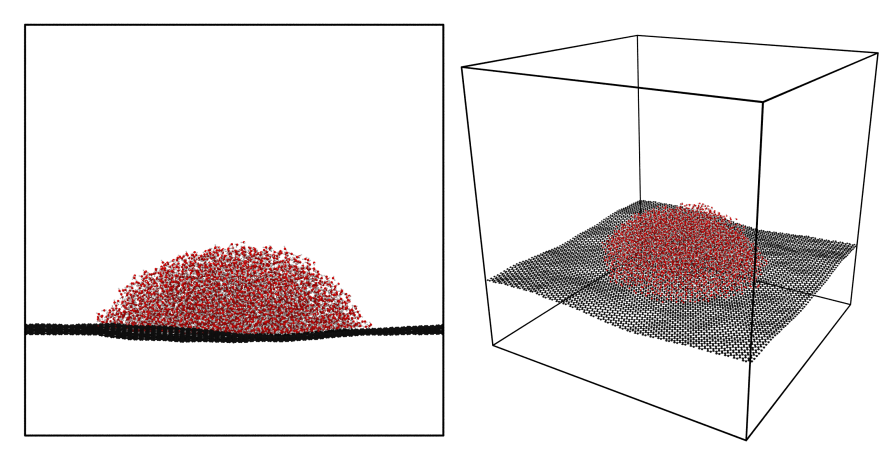
Droplets on fixed graphene (154.0 Å × 148.2 Å ×151.1 Å)	$N_{\text{atoms}} \in \{22680; 14640; 11640\}$ $N_{H_2O} \in \{4680; 2000; 1000\}$ $t_{\text{eq}} = 100\,\text{ps}$ $t_{\text{sim}} = 1.20\,\text{ns}$	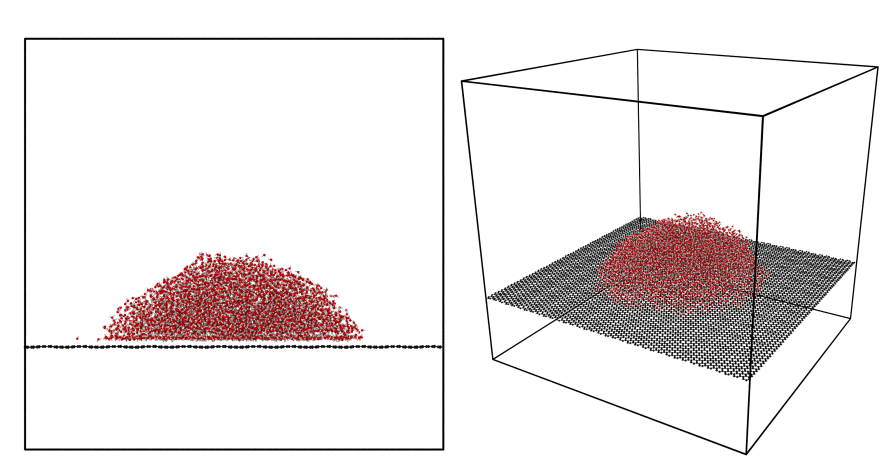

Table S1 (continued)

**System (Dimensions)**	**Simulation details**	**Illustration of largest example**
Droplets on tensile-strained graphene (154.3 Å × 148.5 Å to 157.1 Å × 151.2 Å ×151.1 Å)	$N_{\text{atoms}} = 11640$<br>$N_{H_2O} = 1000$<br>$t_{\text{eq}} = 100\,\text{ps}$<br>$t_{\text{sim}} = 960\,\text{ps}$	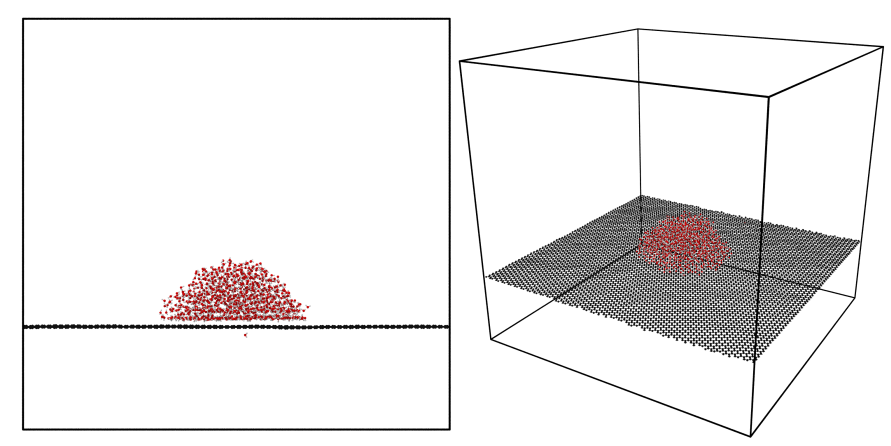
Droplets on fixed, tensile-strained graphene (157.1 Å × 151.2 Å ×151.1 Å)	$N_{\text{atoms}} = 11640$<br>$N_{H_2O} = 1000$<br>$t_{\text{eq}} = 100\,\text{ps}$<br>$t_{\text{sim}} = 960\,\text{ps}$	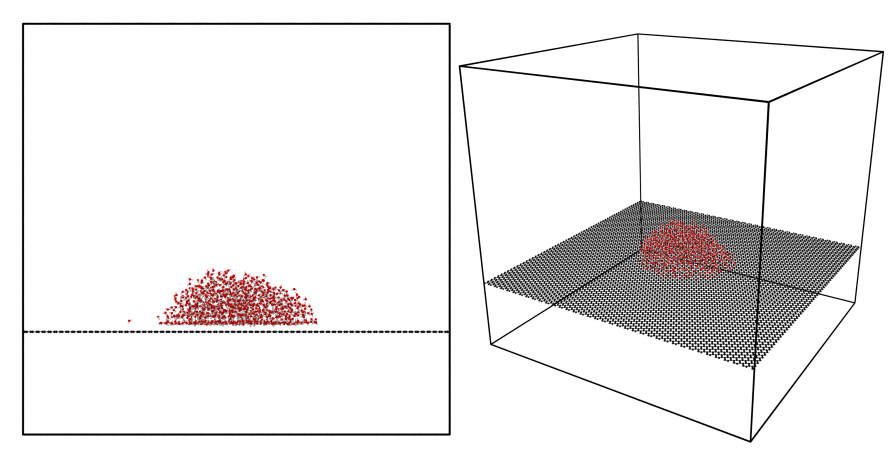
Droplets on compression-strained graphene (150.9 Å × 145.2 Å to 153.9 Å × 148.1 Å ×151.1 Å)	$N_{\text{atoms}} = 11640$<br>$N_{H_2O} = 1000$<br>$t_{\text{eq}} = 100\,\text{ps}$<br>$t_{\text{sim}} = 960\,\text{ps}$	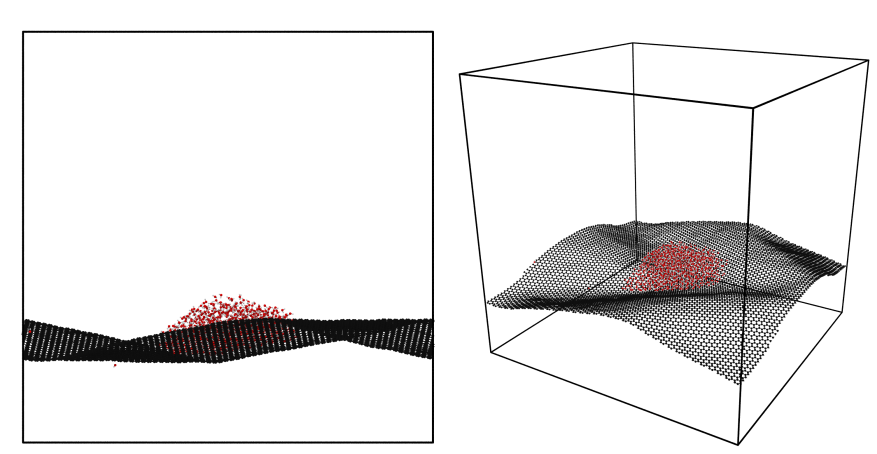

Table S1 (continued)

**System (Dimensions)**	**Simulation details**	**Illustration of largest example**
Free-standing graphene (154.0 Å × 148.2 Å ×151.1 Å)	$N_{\mathrm{atoms}} = 8640$<br>$t_{\mathrm{eq}} = 100\,\mathrm{ps}$<br>$t_{\mathrm{sim}} = 300\,\mathrm{ps}$	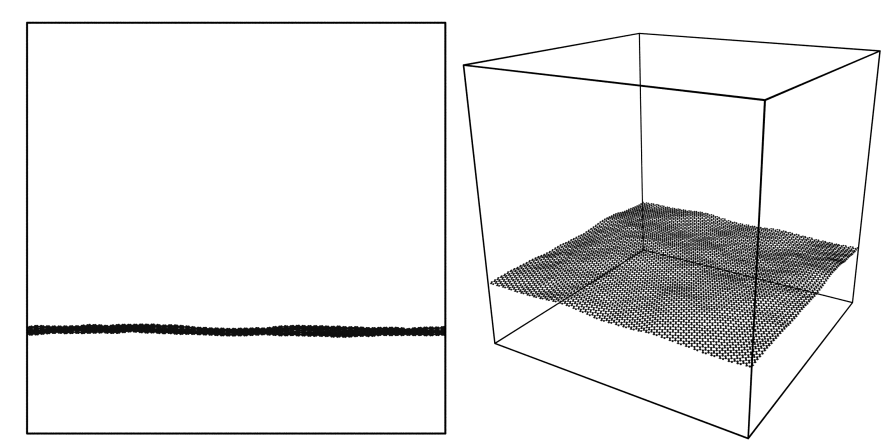
Graphene covered by water (38.5 Å × 37.1 Å ×100.0 Å)	$N_{\mathrm{atoms}} = 4050$<br>$N_{\mathrm{H_2O}} = 1170$<br>$t_{\mathrm{eq}} = 100\,\mathrm{ps}$<br>$t_{\mathrm{sim}} = 300\,\mathrm{ps}$	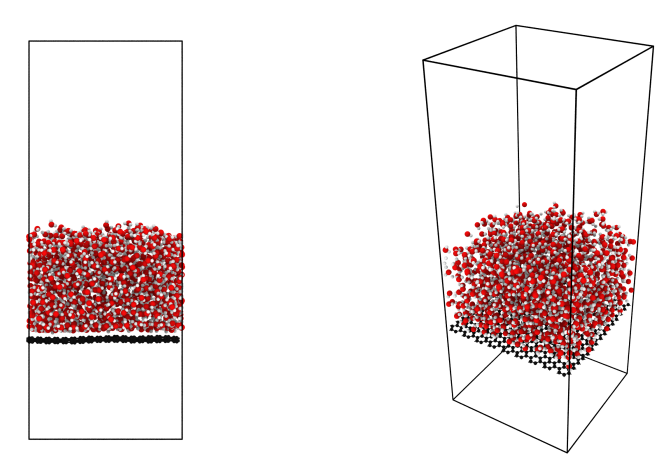
Bulk water, NVT (12.42 Å cubic)	$N_{\mathrm{atoms}} = 192$<br>$N_{\mathrm{H_2O}} = 64$<br>$t_{\mathrm{eq}} = 300\,\mathrm{ps}$<br>$t_{\mathrm{sim}} = 1\,\mathrm{ns}$	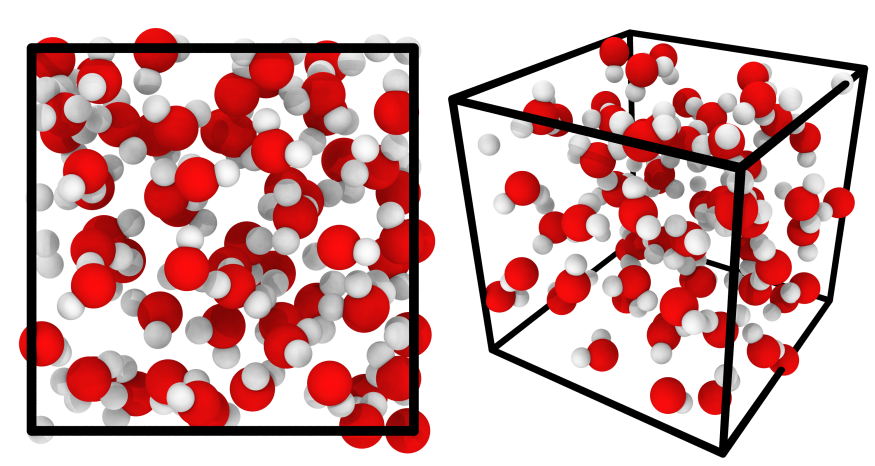
Bulk water, NpT (Average: 25.1 Å cubic)	$N_{\mathrm{atoms}} = 1560$<br>$N_{\mathrm{H_2O}} = 520$<br>$t_{\mathrm{eq}} = 300\,\mathrm{ps}$<br>$t_{\mathrm{sim}} = 1\,\mathrm{ns}$<br>$p = 1\,\mathrm{atm}$	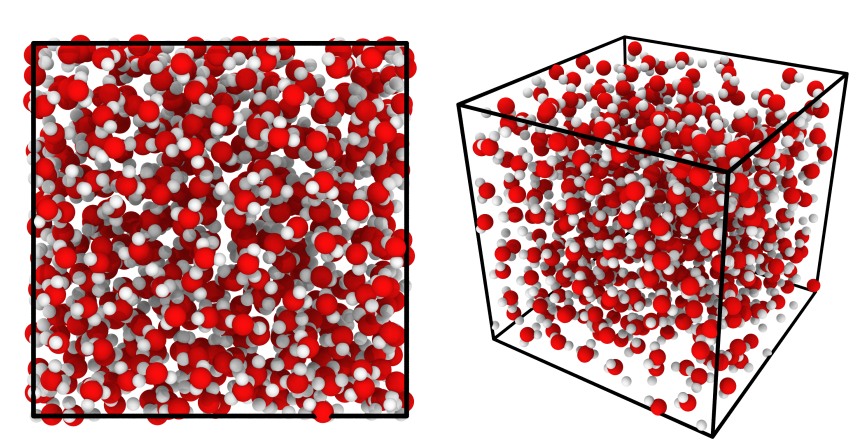

Table S1 (continued)

**System (Dimensions)**	**Simulation details**	**Illustration of largest example**
Water slab (25.2 Å × 25.2 Å to 75.5 Å × 75.5 Å ×75.0 Å)	$N_{\mathrm{atoms}} \in \{14040; 9750; 6240; 3510; 1560\}$ $N_{\mathrm{H_2O}} \in \{4680; 3250; 2080; 1170; 520\}$ $t_{\mathrm{eq}} = 100\,\mathrm{ps}$ $t_{\mathrm{sim}} = 1\,\mathrm{ns}$	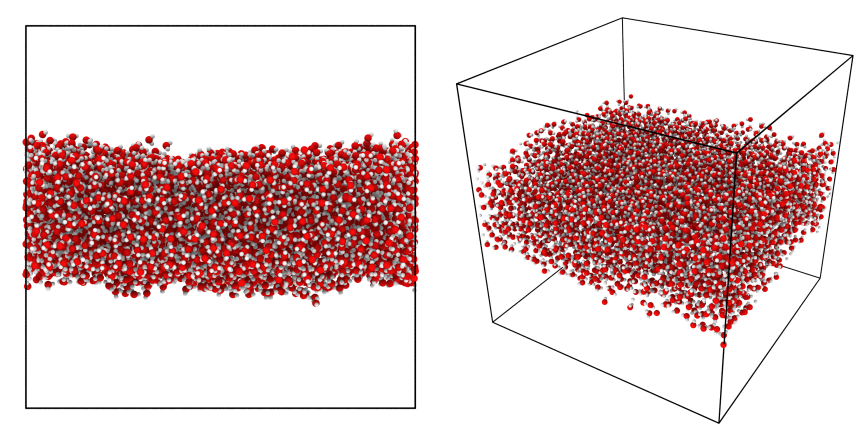

Machine learning potential

The MLP used in this work is the same as that of Ref. S4, where it has already been extensively benchmarked. Here, we briefly recap some of the details regarding the development and validation of this model as reported in Ref. S4, as well as further tests performed for this work. Beyond the validations presented in this section, we also performed additional benchmarks for the density, radial distribution function, and surface tension of bulk liquid water (see next section).

Model development

The MLP was developed iteratively over several generations, beginning with training data from Refs. S5 and S6, selected to provide broad coverage of the relevant physical regimes. These datasets provide (i) self-dissociated water configurations across a wide range of densities under graphene confinement, including the ultra-confined limit, and (ii) water-graphene interfaces spanning from flat sheets to highly curved environments within carbon nanotubes of varying radii. Together, they ensure that the model captures both the physics of graphene-water interactions and the bending rigidity of graphene, which is an essential feature for accurately modelling graphene surface rippling.

Additional structures, involving graphene layers stacked in both the AA and AB configurations at varying interlayer distances, were also introduced in order to refine the description of graphene-graphene interactions, although such interactions are not significant for this work. The training data was also extended to include graphene sheets of large dimensions, to ensure the model's applicability to the large scales demonstrated in this work.

In total, the training set comprised 5,845 structures, which were all labelled with DFT energies and forces computed by the CP2K/Quickstep code using the revPBE-D3 functional.

Model validation

To evaluate the validity of the MLP for the systems studied in this work, we quantified the root-mean-square errors (RMSEs) in energies and forces predicted by the model. Specifically, 300 snapshots were randomly selected from 100 ps MLP-based MD simulations of either bulk liquid water, or monolayer water confined between two 12.350 Å × 12.834 Å graphene sheets. For each configuration, we performed single-point DFT calculations at the same level of theory used to train the MLP (i.e. revPBE-D3). This provides a direct and robust measure of the MLP's accuracy, as it compares predictions for structures outside of the training set sampled from its potential energy surface against the reference ab initio values.

In the case of bulk liquid water configurations, the RMSEs for energies were found to be 0.5 meV/atom, and the RMSEs for forces were found to be 15.4 meV/Å; decomposing these force RMSEs by atomic species gives 21.8 meV/Å for O atoms, and 11.0 meV/Å for H atoms. For the graphene nanoconfined water configurations, the RMSEs for energies were found to be 0.5 meV/atom, and the RMSEs for forces were found to be 26.9 meV/Å; decomposing these force RMSEs by atomic species gives 26.5 meV/Å for C atoms, 35.8 meV/Å for O atoms, and 21.8 meV/Å for H atoms.

Additionally, new previously-unseen test configurations were generated from MD simulations of a liquid water slab on tensile-strained graphene, as shown in Figure S2a. These configurations therefore represent both the water-graphene and water-vapour interfaces. The RMSEs for energies were found to be 0.3 meV/atom, and for forces to be 34.0 meV/Å, with no significant correlations between force errors and DFT-calculated force magnitudes.

Because a central focus of this work is the surface rippling dynamics of free-standing and mechanically strained graphene sheets, it is essential that the model accurately captures the bending rigidity of graphene, which determines how the material deforms. For graphene, the bending rigidity B_M can be obtained by fitting the energy per atom in single-wall carbon nanotubes (SWCNTs) of varying radii r, using the following expression:[S7]

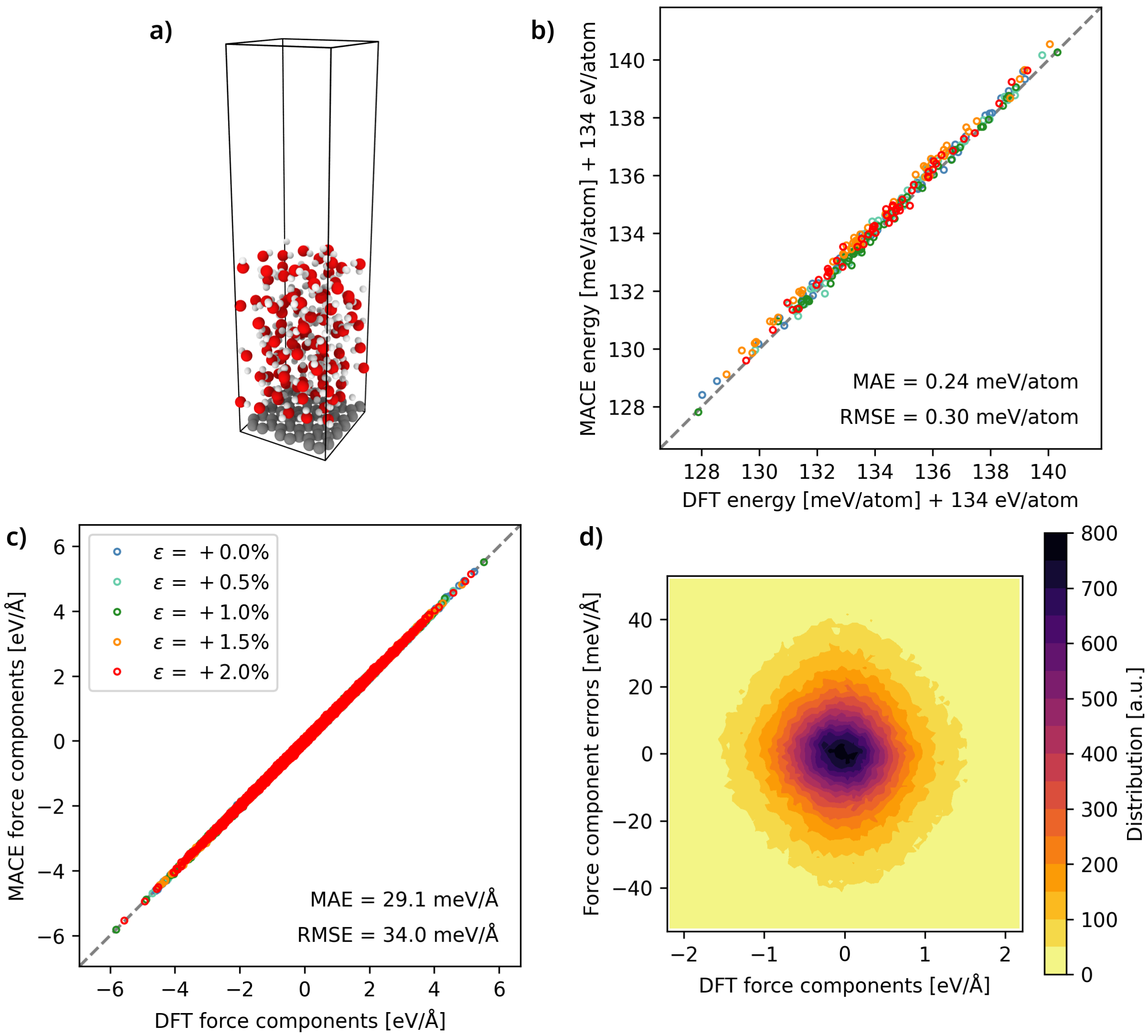

Figure S2: **MLP validation on fully-wetted graphene.** (a) Representative snapshot of one of the previously-unseen test configurations, which were generated from short MLP-based MD simulations, containing both water-graphene and water-vapour interfaces. 50 test configurations each were extracted from 5 strain conditions, ranging from free-standing to $\varepsilon = +2.0\%$. (b) Parity plot of MLP-predicted energies per atom (y-axis) against DFT-calculated reference energies per atom (x-axis); note that these reported energies have been shifted by a flat graphene baseline at the same strain condition, to condense the large spread of energies associated with graphene's elastic modulus. (c) Parity plot of MLP-predicted force components against DFT-calculated force components. (d) Joint distribution of MLP-predicted force component errors against DFT-calculated force components, which shows no significant correlations.

$$E_{\text{atom}}^{\text{CNT}} = E_0 + \frac{S_0 B_M}{2r^2} \tag{S1}$$

where $E_{\text{atom}}^{\text{CNT}}$ is the energy per atom in a SWCNT, E_0 is the energy per atom in flat unstrained graphene, and $S_0 = 2.63\,\text{Å}^2$ is the planar footprint of a carbon atom in graphene. By computing $E_{\text{atom}}^{\text{CNT}}$ for nanotubes with different radii, B_M can be obtained from the curvature dependence. As shown in Figure S3, our MLP accurately reproduces this curvature dependence, in excellent agreement with the reference *ab initio* values.

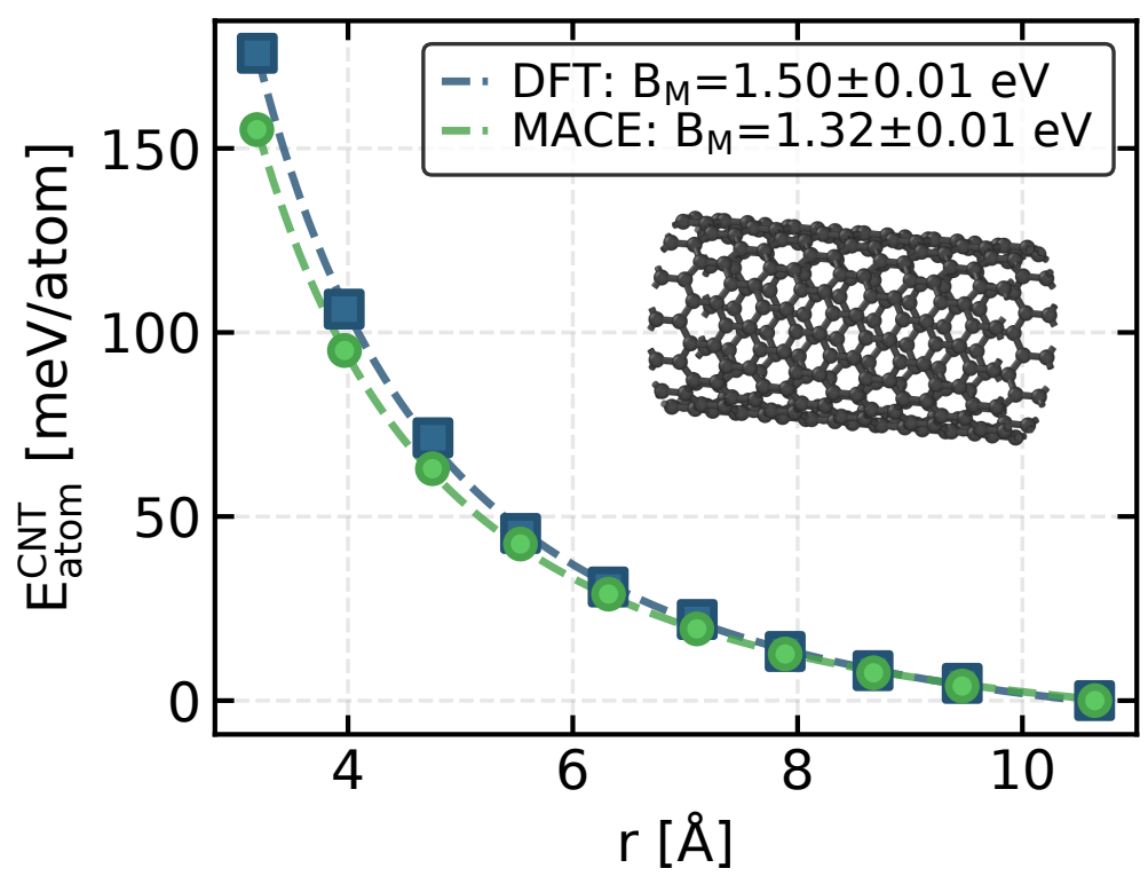

Figure S3: Energy per atom as a function of nanotube radius for SWCNTs rolled along zigzag directions. The dashed line indicates the fit used to extract the bending rigidity B_M of graphene. A representative SWCNT structure is shown as an inset.

Furthermore, to verify that the MLP's description of the potential energy surface at the three-phase contact-line is accurate, additional test configurations were generated from MLP-based MD simulations of a miniaturized water droplet, containing 130 water molecules, placed on a small $51.336\,\text{Å} \times 49.4\,\text{Å}$ graphene sheet under free-standing, $\varepsilon = +2\%$ tensile strain, and $\varepsilon = -2\%$ compressive strain conditions, as shown in Figure S4. The RMSEs for energies were found to be 0.9 meV/atom, and for forces to be 33.2 meV/Å, with no significant correlations between force errors and magnitudes, indicating that the MLP still performs well despite no such droplet configurations being in the training dataset.

Overall, the validations presented in this section demonstrate that the MLP developed in this work reliably captures the physics of water-water and water-graphene interactions, and

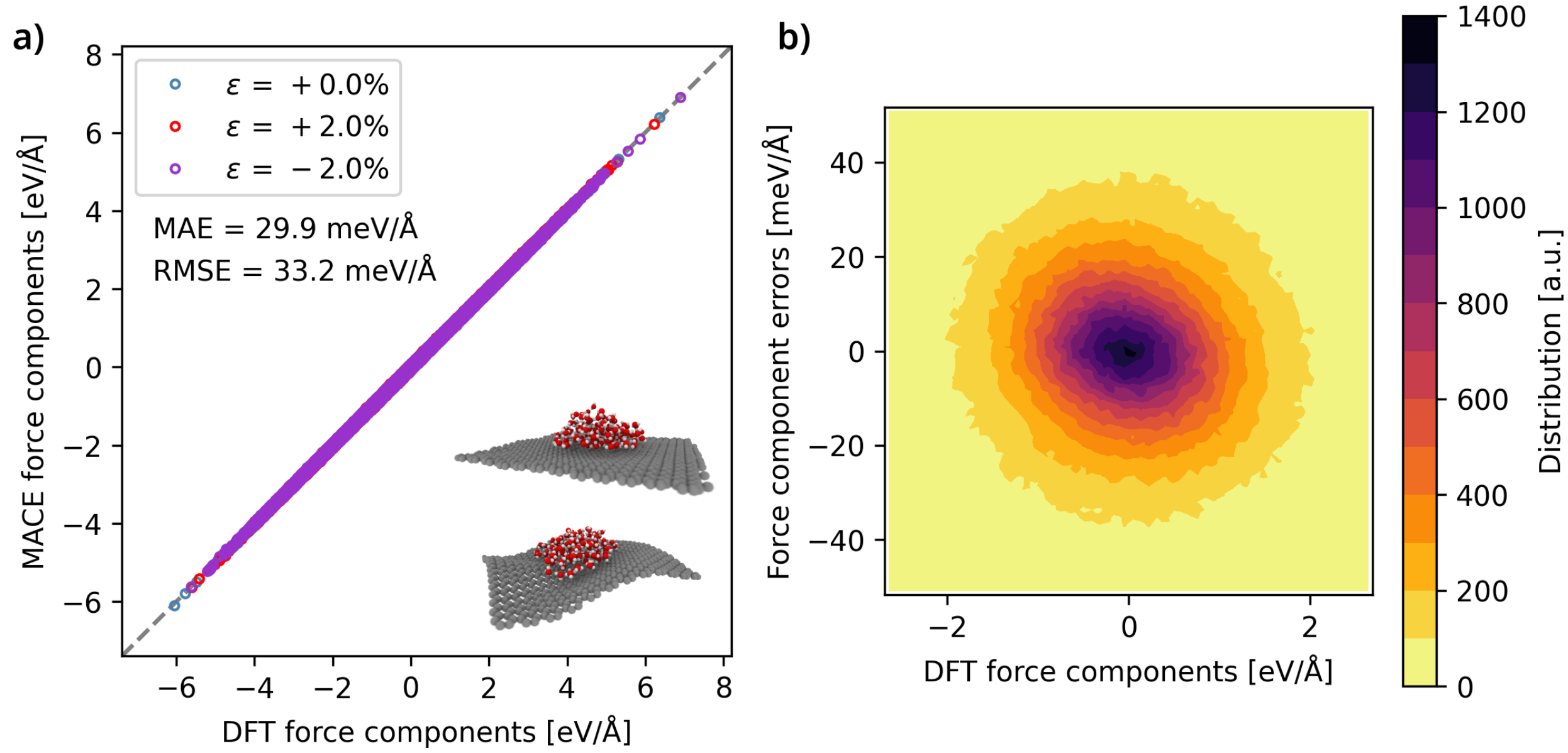

Figure S4: **MLP validation on miniaturized droplets.** (a) Parity plot of MLP-predicted force components (y-axis) against DFT-calculated reference force components (x-axis) for randomly-selected configurations of a small droplet on a graphene sheet under free-standing, tensile strained, and compressive strained conditions. Representative snapshots of the free-standing and compressive strained conditions are shown as insets. (b) Joint distribution of MLP-predicted force component errors against DFT-calculated force components.

also the mechanics of graphene bending. With its energetic and force accuracy across a range of conditions, and uniform applicability to both water-water and water-carbon and carbon-carbon interactions, the MLP provides a robust and transferable framework for investigating the wetting of graphene with first-principles level accuracy.

Properties of liquid water predicted by the MLP model

Density and radial distribution function

The O-O radial distribution function (RDF) for bulk liquid water under NVT conditions, as obtained by simulating a cubic 12.42 Å box of 64 water molecules at 300 K for 1 ns with fully periodic boundary conditions using our MLP model, is shown in Figure S5. We compare this directly against an *ab initio* molecular dynamics (AIMD) simulation using the revPBE-D3 functional of the same configuration by Marsalek and Markland,[S8] and find that the structure of bulk liquid water predicted from our MLP model matches that of reference DFT calculations.

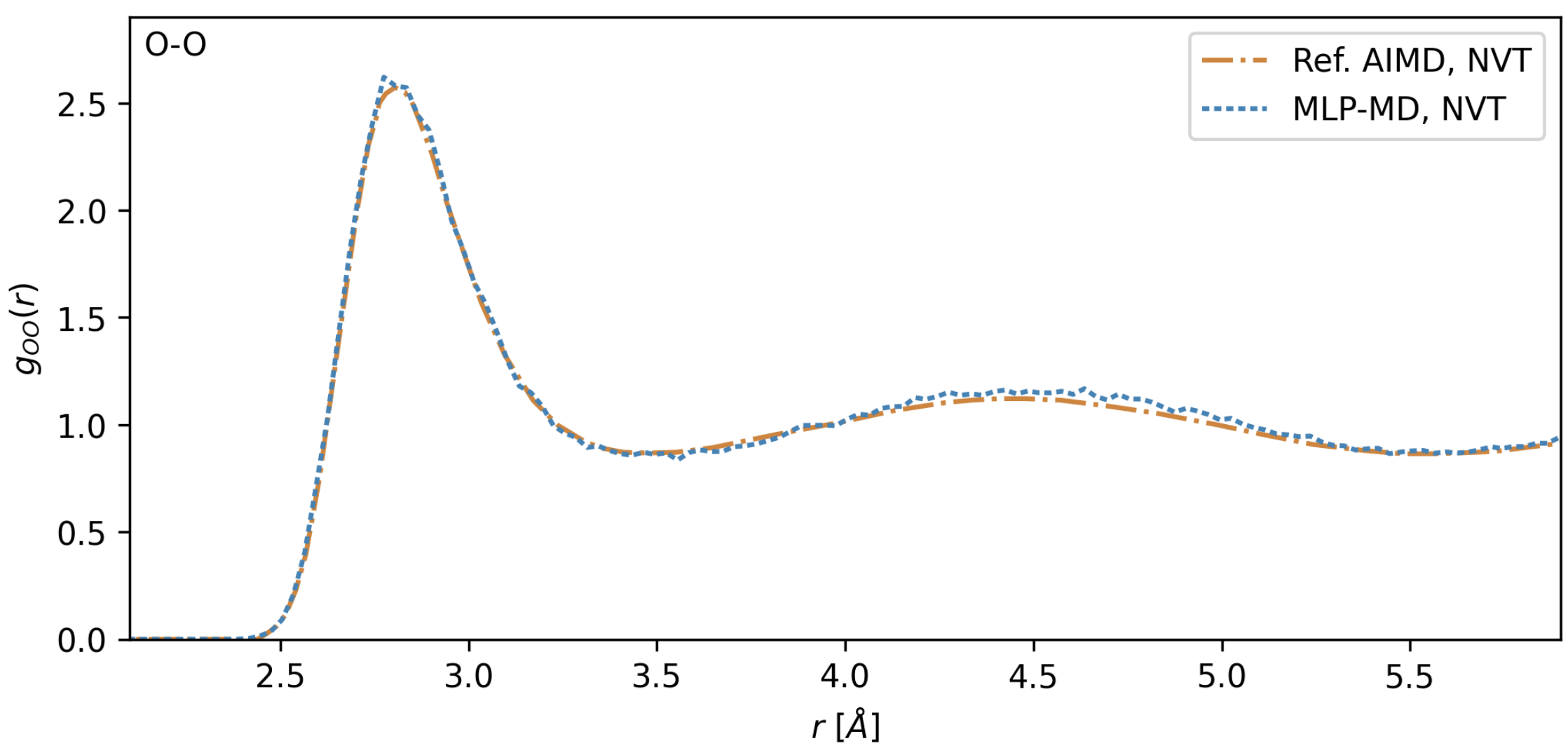

Figure S5: **The radial distribution function (RDF) for O-O interatomic distances in bulk liquid water** under NVT conditions, for a cubic 12.42 Å box of 64 water molecules at 300 K. Dash-dotted gold line: AIMD reference for $g_{\mathrm{OO}}(r)$ using revPBE-D3, from Marsalek and Markland.[S8] Dotted blue line: our MLP model.

Furthermore, to obtain the equilibrium density of bulk liquid water under ambient conditions, a cubic box of 520 water molecules with fully periodic boundary conditions was simulated in the NpT ensemble at a pressure of 1 atm and temperature of 300 K; the pressure condition was maintained isotropically using a Nosé–Hoover barostat with damping time 1 ps. We find that the equilibrium density predicted by our MLP model to be $981.6 \pm 0.7\,\mathrm{kg/m^3}$.

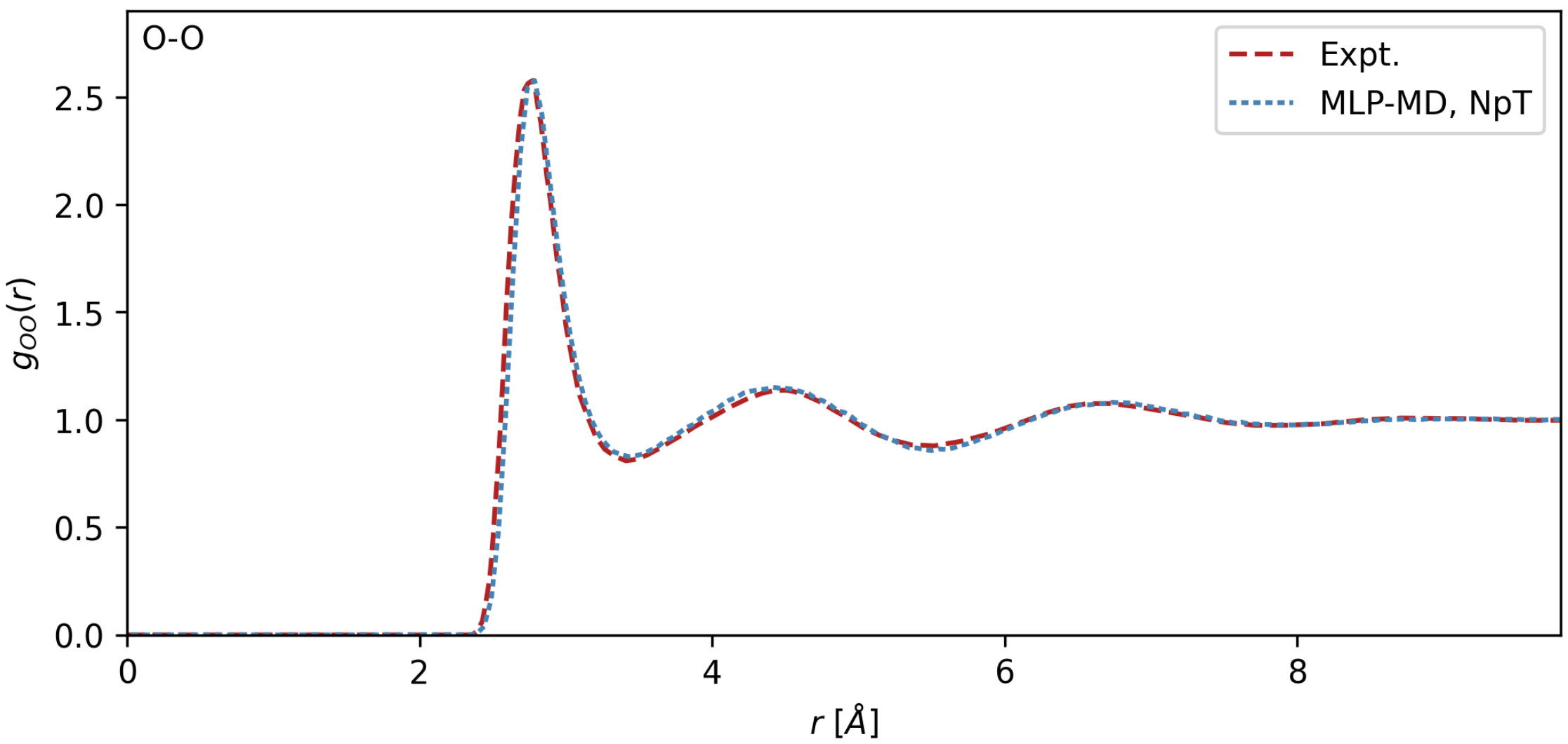

Figure S6: **The RDF for O-O interatomic distances in bulk liquid water under NpT conditions**. Dashed pink line: experimental references from Soper,[S11] based on joint X-ray and neutron diffraction data. Dotted blue line: predicted RDFs from our MLP model, obtained from a simulation of a cubic box of 520 water molecules in the NpT ensemble at $p = 1\,\text{atm}$ and $T = 300\,\text{K}$.

This is within error margin of the reference value of $962 \pm 29\,\text{kg/m}^3$ found by Galib et al.[S9] using an AIMD simulation with revPBE-D3, and also within 2% of the experimental value of $996.5\,\text{kg/m}^3$.[S10]

The RDF for the O-O interatomic distances from this NpT bulk liquid water simulation are also plotted in Figure S6, and compared against an experimental reference measured using joint X-ray and neutron diffraction data.[S11] It is seen that the O-O RDF obtained from the MLP model almost matches the experimental reference, except for a slight shift towards larger r corresponding exactly to the slightly lower predicted density.

In summary, we see that the structure of bulk liquid water as predicted by the MLP model, in terms of both the equilibrium density and the RDFs, closely match the reference DFT calculations. Furthermore, they accurately replicate realistic behaviour as compared to experimental values.

Surface tension

The surface tension of the liquid-vapour interface γ_{lv} is a key parameter in wetting behaviours and the contact angle, e.g. via the Young equation. As such, it is also an important property pertaining to liquid water that should be validated. The surface tension was measured by simulating free-standing slabs of water under the NVT ensemble, spanning the x and y directions with periodic boundary conditions, and extensive vacuum in the z direction; due to the broken translational symmetry along the z-axis, the asymmetrical part of the pressure tensor can be attributed to the surface tension of the liquid-vapour interface as follows:

$$\gamma_{\mathrm{lv}} = \frac{L_z}{2}\left(p_{zz} - \frac{p_{xx} + p_{yy}}{2}\right) \tag{S2}$$

where L_z is the length of the simulation box in the z-axis, and $p_{\alpha\beta}$ are the components of the pressure tensor. A factor of half is inserted due to the two-sided interface of the slab.[S12,S13]

Due to finite-size effects, the measured surface tension in these simulations are affected by the length of the simulation box $L_{\parallel}$ along the x and y directions, as the periodic boundary conditions constrain the spectrum of surface capillary waves allowed at the interface. This can be compensated for using a finite-size correction of the form:

$$\gamma_{\mathrm{lv}}(L_{\parallel}) \approx \gamma_{\mathrm{lv},\infty} - k\,\frac{\ln L_z}{{L_{\parallel}}^2} \tag{S3}$$

for some arbitrary fitting constant k, where $\gamma_{\mathrm{lv},\infty}$ is the macroscopic surface tension.[S14–S16]

To perform this finite-size correction, we simulated five free-standing water slabs of between 520 to 4,680 water molecules, with width $L_{\parallel}$ ranging from 25.18 Å to 75.54 Å. The thicknesses of the water slabs were seen to equilibrate at around ∼25 Å for all five slabs. We find the macroscopic surface tension to be $\gamma_{\mathrm{lv},\infty} = 74.5 \pm 1.4$ mN/m, which differs by no more than 4% from the known experimental value of 71.7 ± 0.4 mN/m.[S17] It is also within the error margin of the reference value of 83 ± 28 mN/m obtained from AIMD using the same revPBE-D3 level of theory,[S16] although the error margin of this AIMD study is large due to

high computational costs limiting the achievable simulation timescales. This gives further confidence in the MLP model's ability to simulate the liquid-vapour interface accurately.

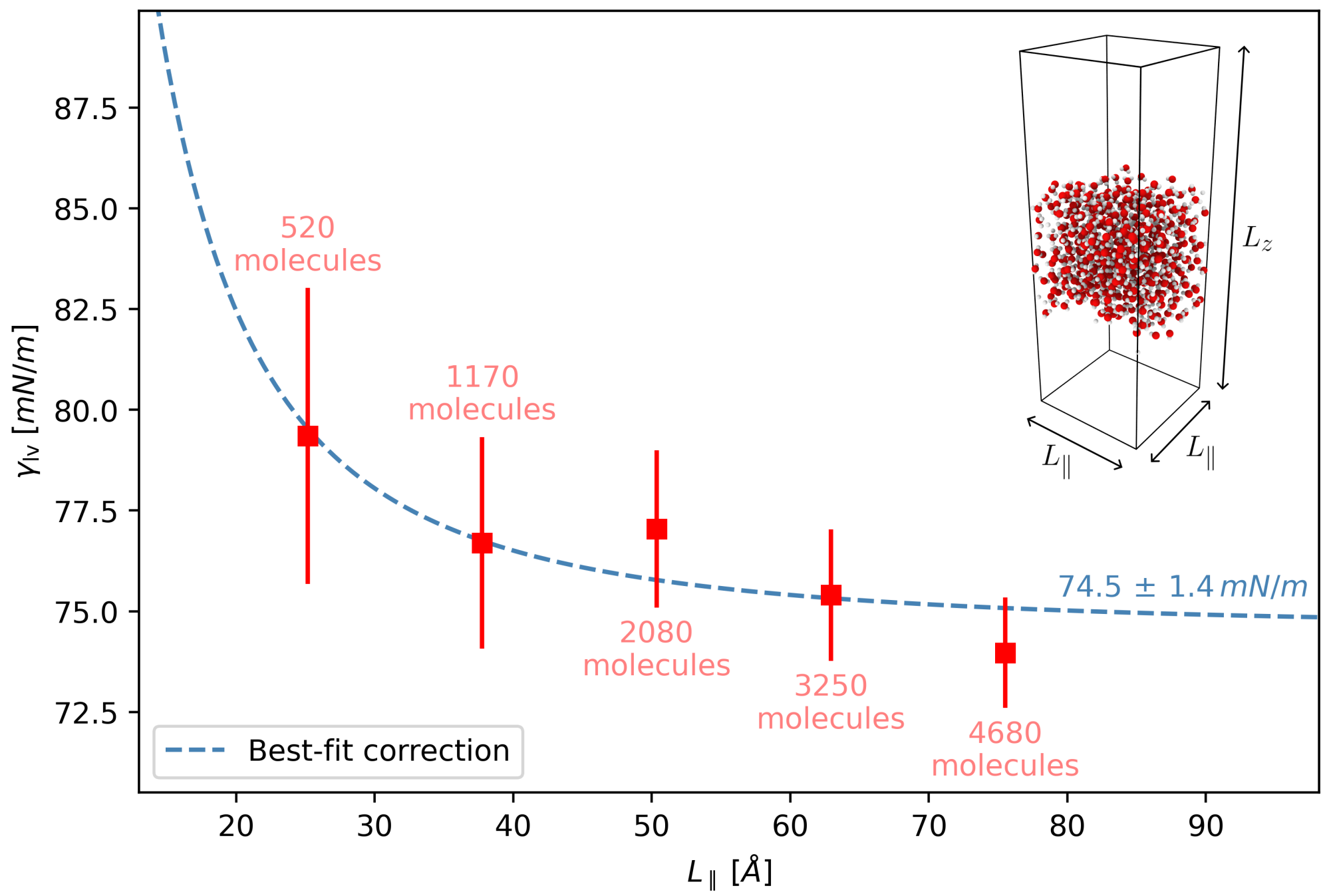

Figure S7: **The liquid-vapour surface tension of water** at 300 K as obtained from the MLP model, across varying sizes of water slabs; the inset shows a representative frame of the smallest slab, and the dashed blue line represents the best-fit finite-size correction following eq. (S3). Extrapolating this correction yields a macroscopic surface tension of $\gamma_{\text{lv},\infty} = 74.5 \pm 1.4\,\text{mN/m}$.

Code implementation for obtaining the contact angle on non-flat surfaces

The methodology used to obtain the contact angle throughout this work, as described in Methods, was implemented in Python for ease of development and portability. The code has been deposited in GitHub (https://github.com/fast-group-cam/contact-angle).

We use the coordinate convention, where the graphene sheet is nominally aligned to the xy-plane (i.e. normal to the z-axis), and atomic coordinates are re-centred on every timestep such that the mean oxygen x- and y-coordinates are both zero and the mean carbon z-coordinate is zero at all times t. Note that this coordinate re-centring is only applied to the analysis of contact angles and graphene sheet dynamics from the simulation trajectories, and not to the simulations themselves.

Algorithm for determining the time-averaged interface

For a simulation propagated over discrete timesteps $t \in \{t_1, t_2, \ldots\}$, the time-averaging of the coarse-grained density field eq. (2) is carried out in practice by summing over the frames:

$$\begin{aligned} \langle \bar{\rho}(\mathbf{r}) \rangle_t &:= \frac{1}{T} \int_0^T \bar{\rho}(\mathbf{r}, t) \, \mathrm{d}t \\ &\approx \frac{1}{N_t} \sum_{\{t\}} \sum_{i=1}^{N_{\text{oxy}}} \left(2\pi\xi^2\right)^{-3/2} \exp\left[-\frac{|\mathbf{r} - \mathbf{R}_i(t)|^2}{2\xi^2} \right] \end{aligned} \tag{S4}$$

for a fixed coarse-graining length $\xi = 2.4$ Å. The value of the time-averaged coarse-grained density field at any point $\mathbf{r}$ can be calculated efficiently in Python given the array of oxygen positions $\mathbf{R}_i(t)$, using `numpy`[S18] broadcasting and summation.

The time-averaged interface is then the 2-dimensional isosurface $\mathbf{r} = \mathbf{s}$ where the time-averaged coarse-grained density field reaches the cut-off value $\langle \bar{\rho}(\mathbf{s}) \rangle_t = c$, which is set to 0.016 Å^{-3}. This isosurface can be searched for using any root-solving algorithm; a common method to do this, e.g. as implemented by Willard and Chandler,[S19] is to discretize space

into a grid of points, calculate the density at every gridpoint, and identify points on the boundary of $\bar{\rho} > c$ and $\bar{\rho} < c$. This "marching squares" algorithm has a computational time complexity of $\mathcal{O}(\delta x^{-3})$ for spatial resolution δx.

In this work, we instead use a binary search algorithm to rapidly converge the position of the interface along a *searching ray* emanating from a *search start point*, since this is more suited to our application. The algorithm is described as follows:

- Given: search start point $\mathbf{a}_0$ which is in the bulk liquid; search direction $\mathbf{d}$, normalized.

1. Calculate search end point $\mathbf{b}_0 = \mathbf{a}_0 + d_{\max}\mathbf{d}$ for a reasonable maximum search distance $d_{\max}$, such that $\mathbf{b}_0$ is guaranteed to be outside the liquid region. This can be $d_{\max} = \max_{i,t}\{\xi + \mathbf{d} \cdot (\mathbf{R}_i(t) - \mathbf{a}_0)\}$.
2. At the kth iteration, calculate the density $\langle\bar{\rho}(\mathbf{m}_k)\rangle_t$ at the midpoint $\mathbf{m}_k = {}^1\!/_2(\mathbf{a}_k + \mathbf{b}_k)$:
   (a) If $\langle\bar{\rho}(\mathbf{m}_k)\rangle_t > c$, the midpoint $\mathbf{m}_k$ is inside the liquid, so set the new start point $\mathbf{a}_{k+1}$ to $\mathbf{m}_k$.
   (b) If $\langle\bar{\rho}(\mathbf{m}_k)\rangle_t < c$, the midpoint $\mathbf{m}_k$ is outside the liquid, so set the new end point $\mathbf{b}_{k+1}$ to $\mathbf{m}_k$.
3. Repeat step 2 until $|\mathbf{b}_k - \mathbf{a}_k|$ is converged to the precision goal δx.

This algorithm's time complexity scales logarithmically as $\mathcal{O}(-\log\delta x)$; throughout this work, we use a precision goal of $\delta x = 0.01$ Å. The output is thus the location of the intersection between the time-averaged interface $\mathbf{s} = \lim_{k\to\infty}\mathbf{m}_k$ and the searching ray emanating from $\mathbf{a}_0$ in direction $\mathbf{d}$.

For N_t timesteps containing N_{oxy} water molecules, the total computational cost of this interface-finding binary search algorithm — if calculating eq. (S4) naïvely — scales as

$\mathcal{O}(-N_t N_{\text{oxy}} \log \delta x)$ in time complexity and $\mathcal{O}(N_t N_{\text{oxy}})$ in memory complexity due to `numpy` broadcasting. Memory requirements may thus exceed hardware capability if attempting to average over too many timesteps. A crucial 'trick' to greatly reduce the memory footprint can be realized by excluding water molecules far away from the searching ray when calculating eq. (S4), since the rapid fall-off of the Gaussian coarse-graining ensures that such molecules will not affect the location of the interface. In practice, this means that the algorithm only calculates $\langle\bar{\rho}(\mathbf{m}_k)\rangle_t$ using a subset of water molecules within some cut-off distance σ of the searching ray. This 'trick' therefore reduces the time complexity of the algorithm to $\mathcal{O}(-N_t\sigma^2 l \log \delta x)$ and the memory complexity to $\mathcal{O}(N_t\sigma^2 l)$, where l is the expected search distance (usually $l \propto N_{\text{oxy}}^{1/3}$), at a cost of introducing an imprecision of roughly $|\Delta\mathbf{s}| \sim \xi \int_{\sigma/\xi}^{\infty} \zeta^2 \exp(-\zeta^2/2)\,\mathrm{d}\zeta$ to the interface location. We find that setting $\sigma = 3.5\xi$ is sufficient to guarantee a numerical inaccuracy of less than 0.01 Å in typical samples of liquid-vapour interfaces.

Best-fit sphere of the faraway time-averaged interface

The definition of the time-averaged interface, as the isosurface of a smooth analytic function, is paradoxically "too well-behaved"; in particular, the time-averaged interface is guaranteed to be a closed surface with no sharp corners, as illustrated in Figure S8, which means that there is no clear choice for *where* the contact angle occurs on the time-averaged interface itself.

Instead, we draw inspiration from experimental techniques. Contact angles are often measured experimentally using optical goniometry, where the geometry of the droplet is imaged using a camera and the tangent lines of the liquid-vapour and solid-liquid interfaces are extrapolated to find the intersection angle.[S20] For the atomistic context, this therefore corresponds to extrapolating the liquid-vapour region of the time-averaged interface past the "rounded" transitional region, and finding the angle at the sharp intersection of the extrapolated liquid-vapour interface and the solid surface.

The liquid-vapour interface is expected to converge, in the long-time limit, to a spherical surface of uniform curvature due to the minimization of surface energy. As such, the chosen extrapolation is a sphere of the form $|\mathbf{s} - \mathbf{s}_0| = R$ for least-squares best-fit parameters $\mathbf{s}_0$ and R, fitted to points $\mathbf{s}$ on the time-averaged interface far away from the graphene surface (and therefore unaffected by the closure of the solid-liquid portion of the interface, or the

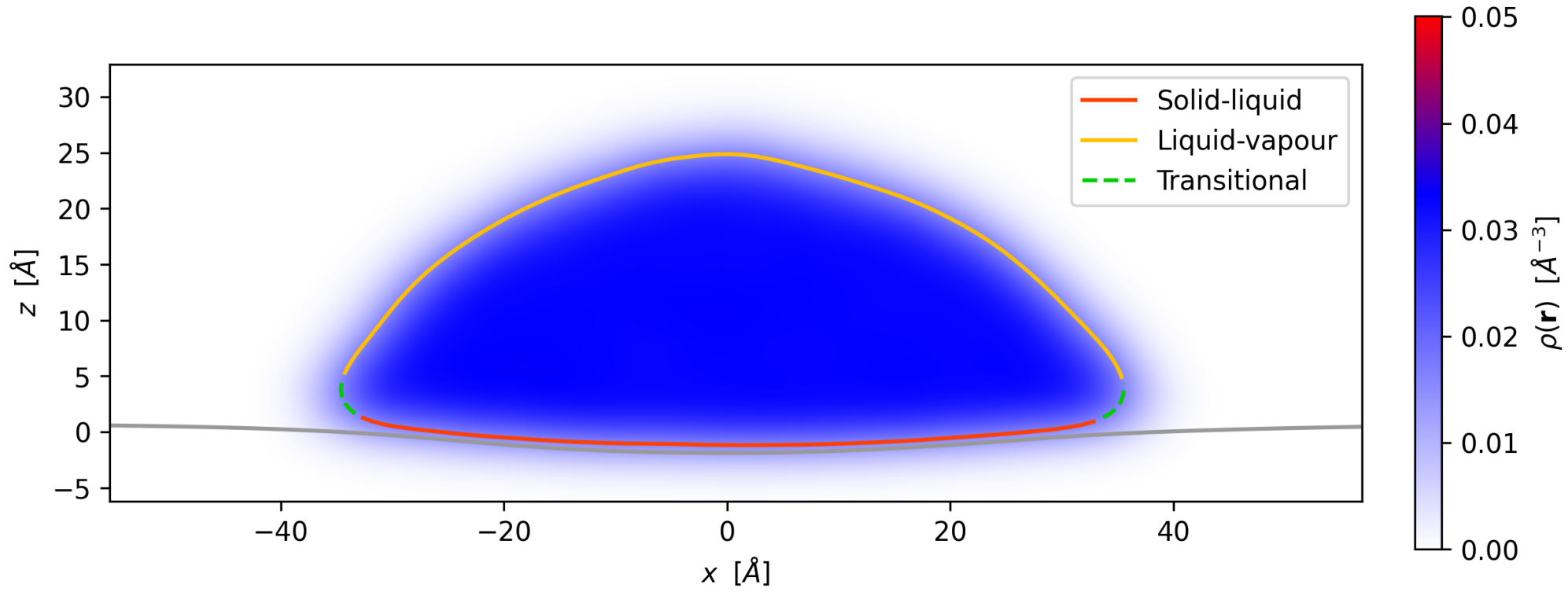

Figure S8: Example plot of the time-averaged coarse-grained density field $\langle\bar{\rho}(\mathbf{r})\rangle_t$, and the time-averaged interface, along the xz-plane for a droplet of 2,000 water molecules. The division of the time-averaged interface into 'solid-liquid', 'liquid-vapour', and 'transitional' regions was performed by eye. In general, the analyticity of $\langle\bar{\rho}(\mathbf{r})\rangle_t$ guarantees that the time-averaged interface is a closed surface with no sharp corners, hence there will always be 'solid-liquid' and 'transitional' regions otherwise irrelevant to the contact angle. Instead, to match experimental definitions, the contact angle should be determined by extrapolating the liquid-vapour region into a sharp intersection with the solid surface (Figure S9).

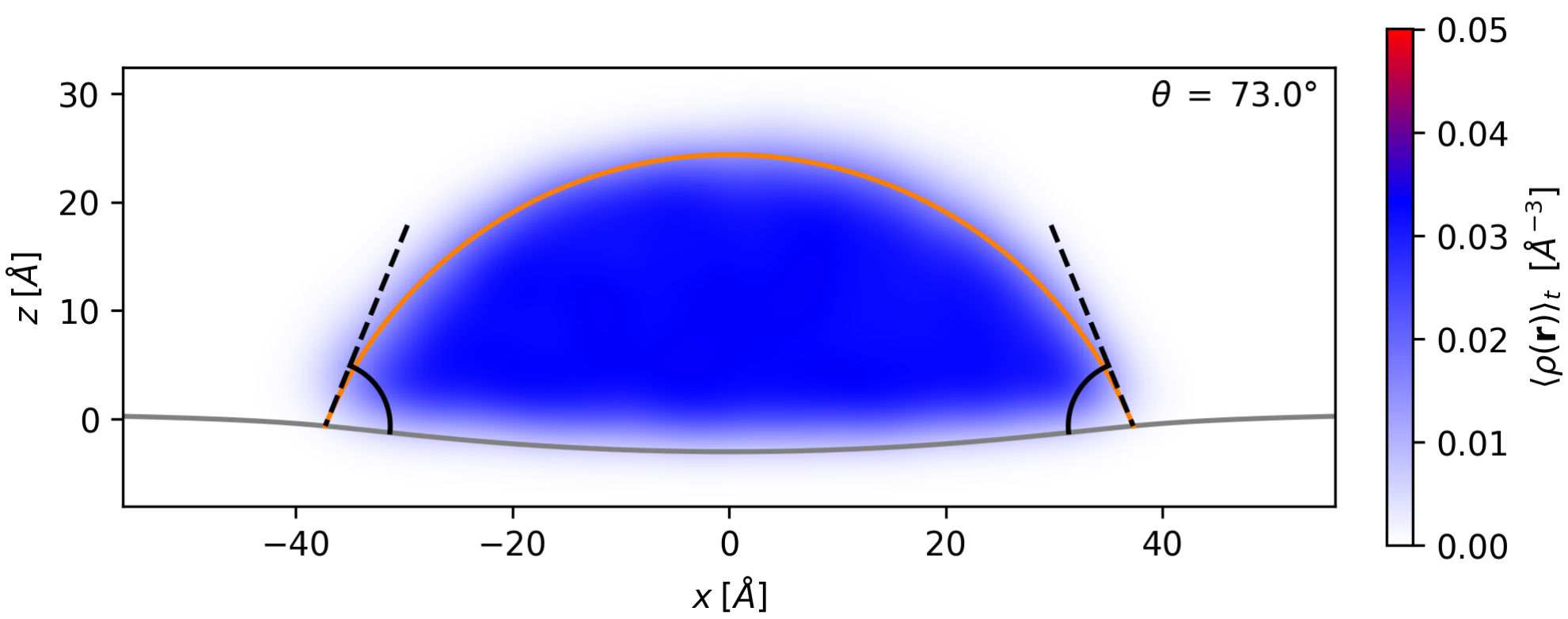

Figure S9: The time-averaged coarse-grained density field $\langle\bar{\rho}(\mathbf{r})\rangle_t$ along the xz-plane for a droplet of 2,000 water molecules (same as Figure S8), and the best-fit sphere (orange line) fitted to randomly-selected interfacial points far from the graphene surface. The contact angle (black lines) is thus defined by the angle between this extrapolated spherical fit and the time-averaged graphene heightmap (dark gray curve).

"rounding" effect of the coarse-graining parameter ξ). These points $\mathbf{s}$ are sampled at random, by generating 150 searching rays emanating from the droplet centre-of-mass's upper hemisphere *not* intersecting the time-averaged graphene heightmap. Specifically, each searching ray is generated by selecting a uniformly-distributed random azimuthal angle between 0 and 2π, and an arccosine-distributed polar angle between 0 and $\frac{\pi}{2}$, and the ray is extended to infinity to check if it collides with the time-averaged graphene heightmap; if no such collision occurs, the interfacial point $\mathbf{s}$ found along this ray is added to the sample, otherwise the ray is re-generated.

For calculating the isotropic contact angle in axisymmetric cases, the centre of the best-fit sphere is constrained to be along the z-axis, so that $\mathbf{s}_0 = (0, 0, z_0)$. Otherwise, for anisotropic contact angles, the centre of the best-fit sphere is fully unconstrained.

The fitting residuals — in this case the distance of the sampled interfacial points $\mathbf{s}$ from the best-fit sphere's surface — are found to have a mean absolute value of 0.11 Å, and a root mean square value of 0.15 Å, when the spherical fit is applied isotropically to a droplet of 1,000 water molecules on free-standing graphene, and a mean absolute value of 0.16 Å and root mean square value of 0.21 Å when the fit is applied anisotropically to the droplet on -2% compressed graphene. For comparison, the best-fit radii for these droplets are 31.43 Å and 34.56 Å respectively, showing that the fitting errors are negligible compared to the scale of the droplet, and that the droplet's faraway liquid-vapour interface is correctly described as a spherical surface in both isotropic and anisotropic cases.

Finding the graphene heightmap

The instantaneous graphene heightmap $h_{\mathrm{GS}}(x, y; t)$ is obtained by constructing a Clough–Tocher interpolator using `scipy`'s built-in `CloughTocher2DInterpolator` constructor,[S21] feeding the x- and y-coordinates of the carbon atoms at time t as the input data and the z-coordinates as the target, and then evaluating the interpolator over a discrete grid of (x, y) points. To handle periodic boundary conditions correctly, the input data to the interpolator

is padded with periodic images in the x and y directions. The bottleneck in this process is the construction of the triangulation mesh for the Clough–Tocher interpolator, which scales as $\mathcal{O}(N_{\mathrm{carbons}})$ independently of the grid resolution.

The discretization of the grid is chosen to always be smaller than the carbon-carbon interatomic distance 1.426 Å, so that the instantaneous heightmap forms a smooth interpolation *within* the hexagonal cells of the graphene sheet. Afterwards, calculating the time-averaged graphene heightmap $\langle h_{\mathrm{GS}}(x,y)\rangle_t$ is a simple matter of averaging the instantaneous heightmaps across the simulation timesteps.

Calculating the anisotropic contact angle

In the general anisotropic case, to find the contact angle along an azimuthal direction ϕ, we first solve for the intersection between the best-fit sphere of the faraway time-averaged interfaces, and the time-averaged graphene heightmap (TAGH):

$$\langle h_{\mathrm{GS}}(x,y)\rangle_t \;=\; z_0 \,+\, \sqrt{R^2 \,-\, (x-x_0)^2 \,-\, (y-y_0)^2} \tag{S5}$$

where $\mathbf{s}_0 = (x_0, y_0, z_0)$ and R are the centre and radius of the best-fit sphere respectively; this equation can be solved 1-dimensionally over variable r where $(x,y) = (r\cos\phi, r\sin\phi)$, using a numerical iterative approach. The anisotropic contact angle along direction ϕ is then:

$$\theta(\phi) \;=\; \arctan\left(\frac{r - x_0\cos\phi - y_0\sin\phi}{\sqrt{R^2 \,-\, (x-x_0)^2 \,-\, (y-y_0)^2}}\right) + \arctan\left(\frac{\partial\langle h_{\mathrm{GS}}\rangle}{\partial x}\cos\phi \,+\, \frac{\partial\langle h_{\mathrm{GS}}\rangle}{\partial y}\sin\phi\right). \tag{S6}$$

This is the approach used to obtain the distribution of anisotropic contact angles (mapped to uniformly distributed ϕ) for droplets under compressive strain. We sample 360 evenly-spaced values of ϕ, finding that this is sufficient to resolve $\theta(\phi)$ continuously.

Calculating the isotropic contact angle

In axisymmetric cases, it is more useful to calculate the isotropic contact angle from the azimuthally-averaged TAGH, which is a function of radial coordinate only:

$$\langle h_{\mathrm{GS}}(r)\rangle_t \;=\; \frac{1}{2\pi}\int_0^{2\pi} \langle h_{\mathrm{GS}}(x = r\cos\phi, y = r\sin\phi)\rangle_t \,\mathrm{d}\phi \tag{S7}$$

in which case the intersection with the best-fit sphere of the faraway time-averaged interface is simply the solution to:

$$\langle h_{\mathrm{GS}}(r)\rangle_t \;=\; z_0 + \sqrt{R^2 - r^2} \tag{S8}$$

and the solution $r = a$ can be interpreted as the radius of the three-phase contact line. The isotropic contact angle is then:

$$\theta \;=\; \arctan\left(\frac{r}{\sqrt{R^2 - r^2}}\right) + \arctan\left(\frac{\mathrm{d}\langle h_{\mathrm{GS}}\rangle}{\mathrm{d}r}\right). \tag{S9}$$

This is the approach used to obtain the isotropic contact angles, and corresponding droplet radii, both for the droplets of varying size (to obtain the finite-size corrected contact angle); and also for the droplets under tensile strain.

Parameter sensitivity check

Our contact angle methodology relies on two fixed parameters, the coarse-graining length ξ and cut-off density c, which we set at 2.4 Å and 0.016 Å^{-3} respectively to match the original Willard–Chandler definition.[S19] The coarse-graining length represents the scale of spatial "blurring" required to restore continuity of the density field $\bar{\rho}(\mathbf{r}, t)$ over short sampling times, with the exact equilibrium density field being recovered under the dual limit of $\xi \to 0^+$ and infinite sampling time. On the other hand, the cut-off density represents the threshold for deciding whether a region of space is in the liquid or vapour phase, and therefore must be bounded from below by zero (otherwise "everywhere" is liquid) and from above by the bulk

liquid density (otherwise "everywhere" is vapour). The cut-off density should be set to half of the bulk liquid density, for the time-averaged interface to converge to the Gibbs equimolar dividing surface in the limit of $\xi \to 0^+$.

As a quick check of the sensitivity of our reported contact angles on the methodology's fixed parameters, we performed our analysis for the droplet of 1,000 water molecules on free-standing graphene with varying values of ξ and c, as shown in Figure S10. Both the contact angle and three-phase contact line are not sensitive to changes in the coarse-graining length, as its effect is to suppress spatial fluctuations of the droplet's density field without changing its long time average behaviour. In fact, this hints towards future room for improvement, where the coarse-graining length $\xi(T)$ could be dynamically tuned according to the time-averaging interval T to smoothly connect the instantaneous Willard–Chandler parameter $\xi(T = 0) = 2.4\,\text{Å}$ with the long-time thermodynamic limit $\xi(T \to \infty) = 0$. Nonetheless, Figure S10 shows that our results are robust regardless of how this coarse-graining length is tuned. As for the cut-off density, the contact angle is similarly not sensitive to its value within the neighbourhood of the default value of half the bulk liquid density, but the contact line radius does depend weakly on c, following the geometric intuition that a larger value of c leads to the liquid-vapour interface being identified closer in to the liquid region, thus shrinking the droplet's fitted shape. Indeed, the reported results diverge when the cut-off density is too close to the bulk liquid density, as the liquid region becomes undefined. We therefore recommend that the cut-off density should be kept at $c = \frac{1}{2}\rho_{\text{bulk}}$, to maintain connection with the equimolar dividing surface, noting that the reported contact angles do not depend on this choice anyways.

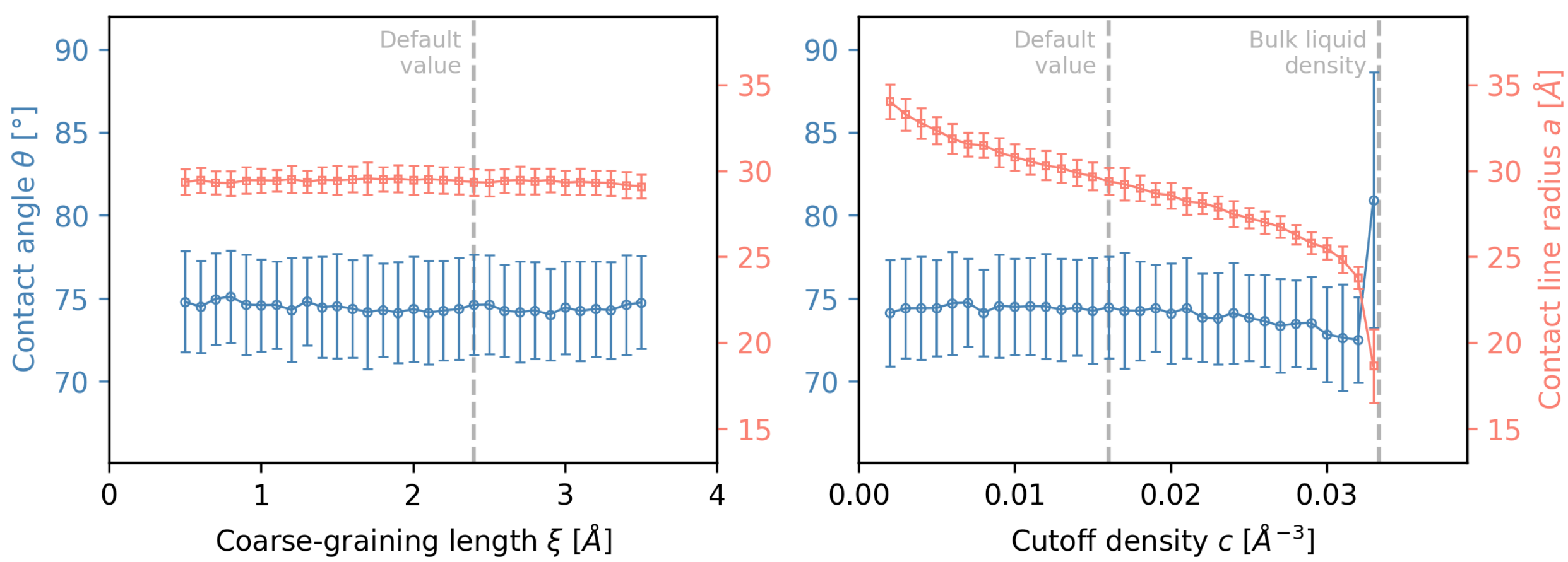

Figure S10: **Sensitivity check of methodology fixed parameters on contact angles and contact line radii.** Left: the microscopic contact angle (blue circle markers) and three-phase contact line radius (red square markers) for a droplet of 1,000 water molecules on free-standing graphene, analysed using varying values of the coarse-graining length ξ. The error bars represent the 95% confidence interval from block averaging. Right: the same results for varying values of the cut-off density c.

Comparison of contact angle methodologies on spatially-fixed flat graphene and dynamical graphene (unstrained)

In order to compare our novel methodology for measuring contact angles against the literature-established methodology, e.g. as detailed by Werder et al. (Ref. S22), we simulated three droplets of different sizes on spatially fixed, flat graphene sheets and measured their contact angles using both methodologies. These separate simulations are necessary, as the Werder et al. methodology is limited to flat solid surfaces by nature of the z-coordinate based histogram. We find that both methodologies give consistent results for the contact angles on these frozen sheets, as shown in Figure S11, verifying that our methodology as introduced in this work is consistent with previous interpretations of the contact angle.

More interestingly, we compare the contact angles obtained for the droplets of the same sizes on dynamical free-standing graphene (also Figure S11), which can only be measured using our methodology. We find that the contact angles on free-standing graphene sheets are within error margin of those on spatially frozen graphene sheets. These findings are in line with the observations reported by both Werder et al.[S22] and Liao et al.[S23] concerning graphene-water contact angles modelled using empirical force fields. This shows that the contact angle is not significantly affected by whether or not the graphene is capable of deforming flexibly, when the sheet is unstrained.

This presents a contrast against the contact angle of the 1,000 water molecules droplet on graphene under tensile strain, which increases with strain more dramatically for dynamical graphene than for spatially fixed graphene. Such an anomalous effect would not have been discovered using only spatially fixed simulations, highlighting the importance of the machine learning potential based simulations to handle both water-carbon and carbon-carbon interactions at the same level of accuracy.

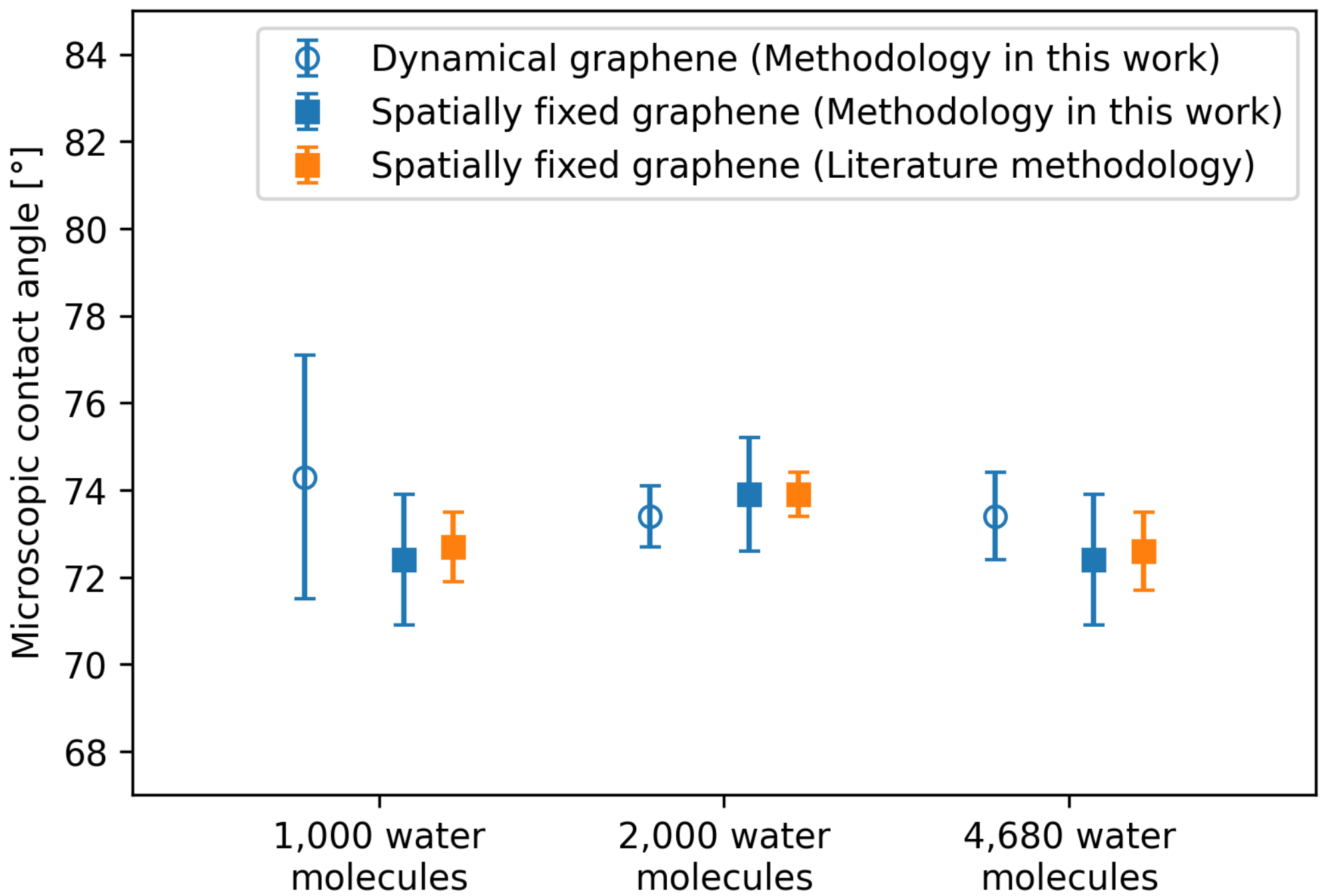

Figure S11: **Contact angles of water droplets of various sizes, as obtained by the MLP, on either dynamical free-standing graphene or spatially fixed flat graphene** with no strain applied. For the fixed sheet, contact angles were measured using both the methodology introduced in this work and also the methodology of Werder et al. (Ref. S22), which gives consistent results. In all three cases, the contact angle on free-standing graphene is within the error margin of the contact angle on spatially-fixed graphene, indicating that the contact angle is not significantly affected by whether or not the graphene is capable of deforming flexibly, as long as the sheet is unstrained.

Interaction energy of water with graphene under tensile strain

The increase of contact angle when tensile strain is applied on the graphene sheet, from 74.3° in the free-standing case up to 84.8° under +2.0% tensile strain, corresponds to a very large decrease of 67% in the magnitude of the interfacial energy difference $\Delta\gamma = \gamma_{\text{sv}} - \gamma_{\text{sl}}$. This decrease is too large to be explained purely by the increased interatomic spacing of the graphene sheet, which would naïvely account for only a ∼4% reduction in the area density of carbon-water interactions. Instead, this reduction of $\Delta\gamma$ must be driven either by a significant change in the solid-vapour surface tension γ_{sv}, which corresponds to the free energy associated with surface deformations of the graphene sheet, or by a significant change in the solid-liquid surface tension γ_{sl}, which corresponds to the interaction energy between the liquid and solid phases.

To clarify which mechanism dominates the drastic decrease in hydrophilicity under tensile stress, we calculate the single-molecule static binding energy between a water molecule and a graphene sheet, as a function of the distance d. The binding energy is defined as:

$$E_b(d) = E_{\text{W+G}}(d) - E_{\text{W}} - E_{\text{G}} \tag{S10}$$

where $E_{\text{W+G}}(d)$ is the total energy of the system where the water molecule is placed at distance d from the graphene sheet, E_{W} is the energy of the isolated water molecule, and E_{G} is the energy of the isolated graphene sheet. Note that this involves only the static electronic energy, without thermal or nuclear quantum effects.

We calculate this binding energy curve $E_b(d)$ for varying water molecule orientations (0-leg, 1-leg, and 2-leg) and tensile strains applied to the graphene sheets, using both the MLP and using DFT calculations at the revPBE-D3 level from CP2K/Quickstep under the same settings as those used to train the MLP. These binding energy curves are plotted in Figure S12. In all cases, the MLP model is able to replicate the DFT calculations closely,

regardless of the orientation of the water molecule or the strain on the graphene sheet. It is also seen that the application of strain does not significantly affect the binding energy curves, for all water orientations.

The effects of strain on the interaction energy between liquid water and the graphene sheet can be quantified through the single-molecule adsorption energy, which is the magnitude of the minimum of the binding energy curve. We find that the DFT calculated adsorption energy, for the 0-leg motif, decreases from 88.7 ± 1.0 meV in the unstrained case to 81.8 ± 1.0 meV under +2.0% tensile strain; and for the 1-leg motif, from 148.7 ± 7.0 meV when

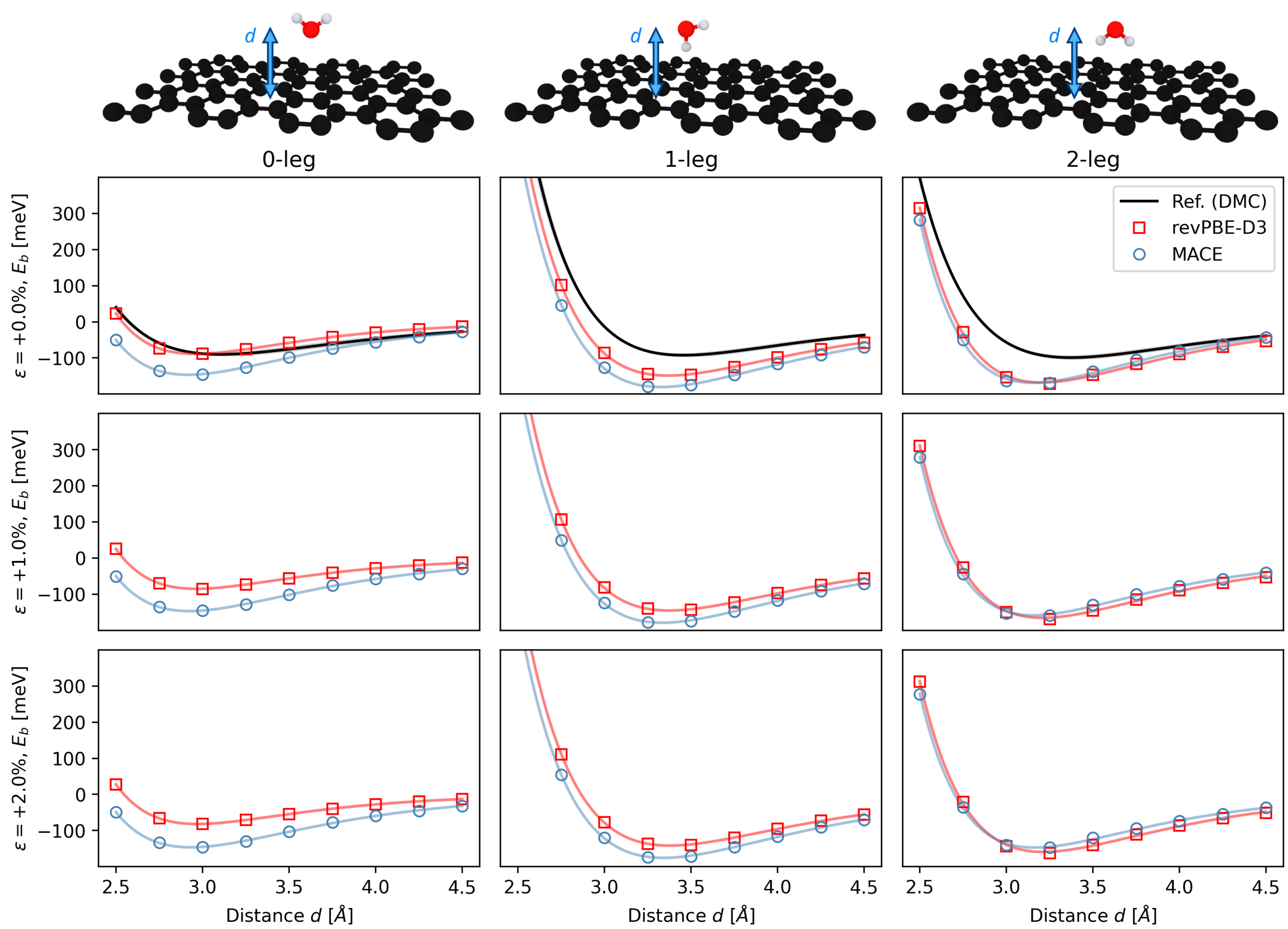

Figure S12: **Single-molecule binding energy curves of water on graphene** in the (left) 0-leg, (middle) 1-leg, and (right) 2-leg motifs, under varying tensile strains ε applied on the graphene sheet from (top) 0% to (bottom) 2%. The red square markers correspond to DFT calculations made using the revPBE-D3 functional in CP2K/Quickstep, while the blue circular markers correspond to the MLP model; the continuous curves are best-fit Morse potentials for each set of calculations. The black curves for the top row for unstrained graphene indicate the reference diffusion Monte Carlo calculations, taken from Ref. S24.

unstrained to 141.6 ± 6.1 meV under +2.0% tensile strain; and for the 2-leg motif, from 168 ± 18 meV when unstrained to 159 ± 19 meV under +2.0% tensile strain. Averaged across all three orientations, the mean adsorption energy thus effectively decreases by only 5.6% when under strain.

As a further test of the effects of strain on the static water-graphene interaction energy, we extracted random configurations of a fully-wetted 12.834 Å × 12.35 Å graphene sheet, with a water slab of ∼20 Å thickness atop, from a thermally-equilibrated MD simulation under varying tensile strain conditions, and computed the binding energy of the entire water slab at its equilibrium distance. The resulting binding energies, as shown in Figure S13, are well-explained by the naïve area-scaling law of $E_b \propto (1+\varepsilon)^{-2}$, which therefore gives only a ∼4% reduction in the water slab's binding energy at 2% tensile strain.

The small decrease in both the single-molecule adsorption energy, on the order of ∼6%,

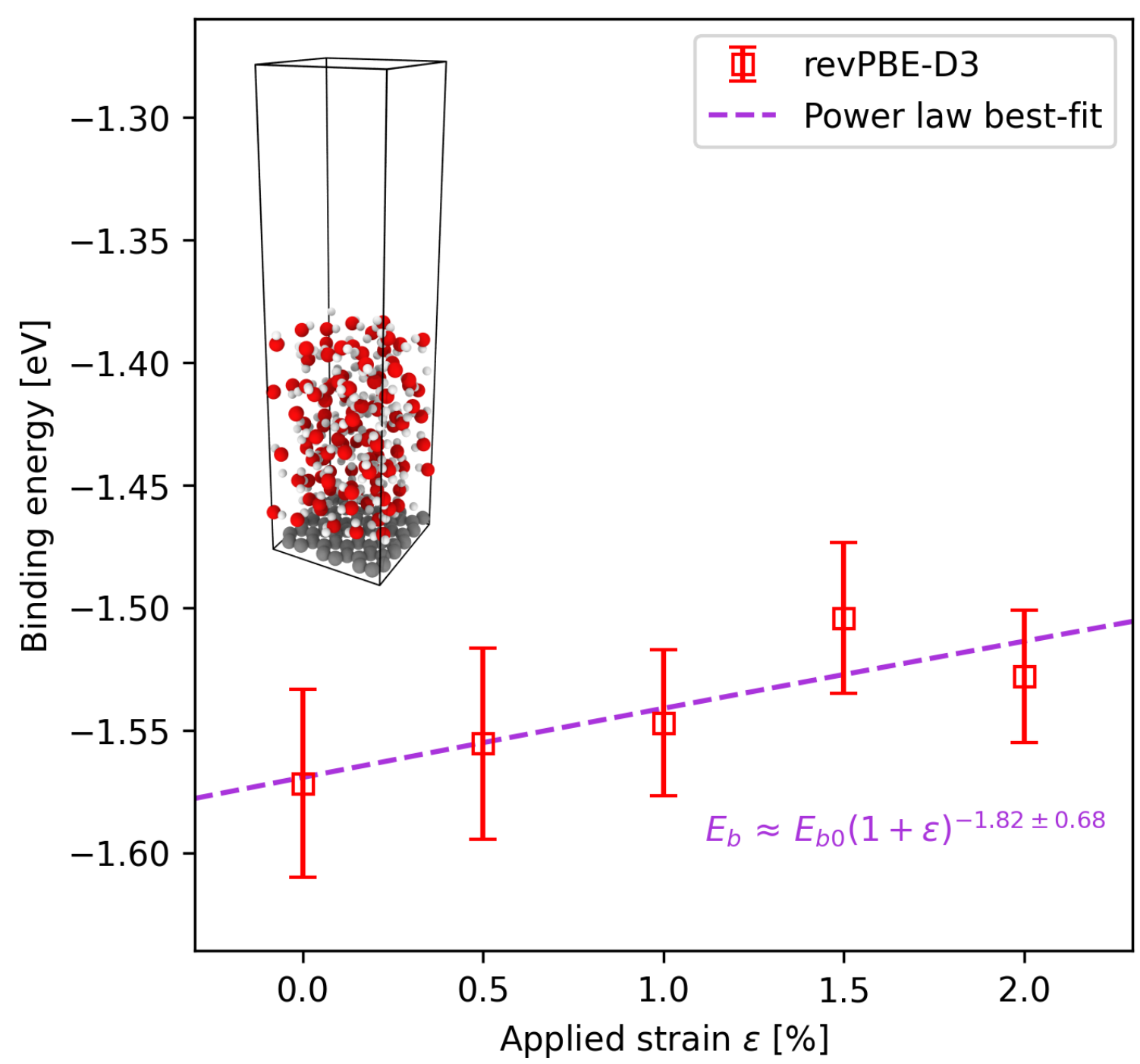

Figure S13: **Binding energy of water slabs on graphene under varying tensile strain conditions,** representing the static DFT interaction energy between water and fully-wetted graphene. The binding energies for each strain condition were averaged over 50 samples randomly selected from a thermally-equilibrated MD simulation, with error bars indicating the 95% confidence interval.

and the water slab binding energy, on the order of $\sim$4%, indicates that the static contribution of the solid-liquid interaction energy likely varies by a similar amount in the original "droplet on graphene" system. Noting that the difference of the solid-vapour and solid-liquid surface tensions $\Delta\gamma$ can be interpreted as a change in the interfacial free energy, which is generally driven by both static interaction energies and entropic contributions from dynamical degrees of freedom, the fact that the solid-liquid interaction energy decreases by a much smaller amount than the 67% decrease of $\Delta\gamma$ indicates that dynamical contributions must drive the strain-modulated weakening of graphene's hydrophilicity. These dynamical contributions may be associated with any part of the system, but the most likely candidate is the free energy associated with surface deformations of the graphene sheet, or in other words by changes in the morphological dynamics of graphene surface rippling, which is illustrated in the localized perturbation of the long-time angle-angle autocorrelation $\mathcal{C}_{\mathrm{GS}}(\tau \to \infty)$ at the droplet edge.

Contact angle on spatially-fixed graphene versus dynamical graphene under +2.0% tensile strain

The evidence of the DFT-calculated interaction energy for a single water molecule upon spatially fixed graphene, which only decreases by 8% when under +2.0% tensile strain, demonstrates that the change in static interaction energies is insufficient to explain the drastic weakening of hydrophilicity of free-standing graphene under tensile strain. To further compare whether or not the observed increase of contact angle under tension is related to the dynamical rippling motion of the graphene membrane, we also simulated a droplet of 1,000 water molecules on a spatially fixed, flat graphene sheet under +2.0% tensile strain.

The resulting contact angle on this spatially-fixed strained sheet is 80.8 ± 1.4°. This represents an increase in the contact angle (i.e. a weakening of hydrophilicity) compared to the free-standing value of 74.3 ± 2.8°, but still significantly less than the contact angle of 84.8 ± 1.2° seen on dynamical graphene under the same tensile strain. In particular,

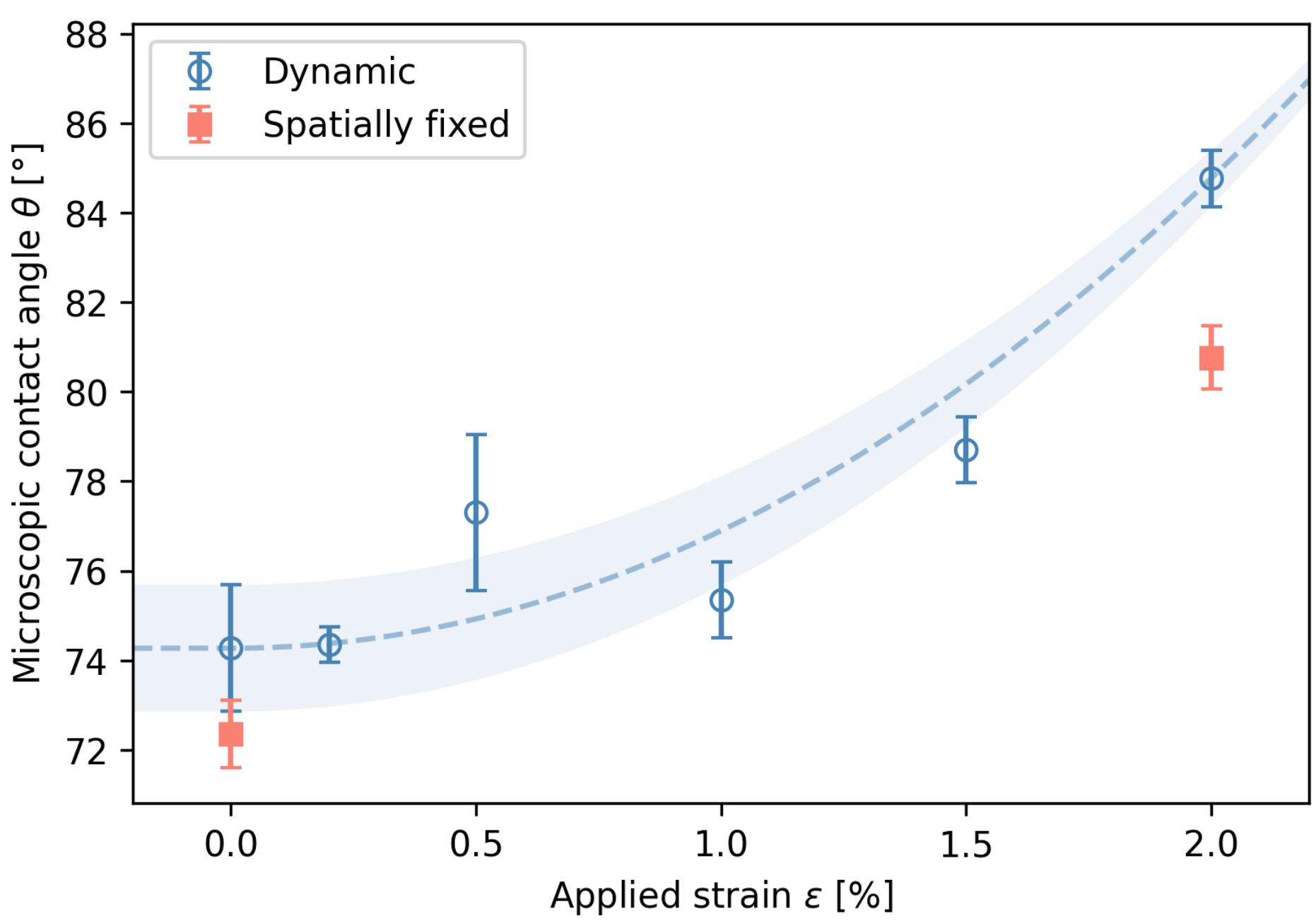

Figure S14: **Contact angles for the droplet of 1,000 water molecules under varying tensile strain, on dynamical versus spatially fixed flat graphene sheets.** The dashed blue line is a guide to the eye, and does not represent any specific functional form.

this implies that the surface energy difference $\Delta\gamma = \gamma_{\text{lv}} \cos\theta$ between the solid-vapour and solid-liquid interfaces is decreased by 41% on spatially-fixed graphene under only a 2% strain, which is surprisingly large but nonetheless insufficient to account for the nearly 67% decrease of $\Delta\gamma$ on dynamical graphene.

These results point towards the idea that the strain-driven modulation of wetting is partially explained by weakening static intermolecular effects, e.g. as supported by the decrease in DFT-calculated single-molecule interaction energy, whose effect can be captured by spatially fixed simulations with first-principles level accuracy; however, the remaining anomalous increase of contact angle is driven from the unique dynamical properties of graphene's surface ripples and their coupling to the three-phase contact line, which is then modified by strain.

“Surfing” motion of droplet on long-ranged coherent ripple waves

The application of compressive strain on a graphene sheet results in a phase transition, where the sheet spontaneously “buckles”, and transitions from thermally random small-amplitude surface ripples to a large-amplitude long-ranged coherent ripple wave. This has been reported in both the experimental and theoretical literature before, e.g. in Refs. S25–S28. For our simulations, we establish the critical point of this phase transition by studying the $(m, n)^{\text{th}}$ Fourier coefficients for the graphene sheet heightmap $h_{\text{GS}}(x, y; t)$, defined as follows:

$$c_{(m,n)}(t) \; = \; \frac{1}{L_x L_y} \int_0^{L_x} \int_0^{L_y} h_{\text{GS}}(x, y; t) \, \exp\left(i \frac{2\pi m}{L_x} x\right) \, \exp\left(i \frac{2\pi n}{L_y} y\right) \mathrm{d}x \, \mathrm{d}y \qquad \text{(S11)}$$

where L_x and L_y are the lengths of the simulation box in the x and y directions respectively. Since the longest-ranged coherent ripple corresponds to the lowest (m, n), the magnitudes of the first Fourier coefficients for each direction $|c_{(1,0)}|$ and $|c_{(0,1)}|$ serve as a useful indicator for the random-to-coherent rippling phase transition. The distribution of these magnitudes is plotted in Figure S15.

The phase transition is marked by a bifurcation of the two modes, which occurs for compressive strains greater than or equal to -0.20%; the dominant $(1, 0)$ mode gains a non-zero time-average, whereas the $(0, 1)$ mode is thermally distributed around zero. Visually, this corresponds to the appearance of a long-ranged coherent ripple wave on the graphene sheet. On the other hand, for all tensile strains, as well as compressive strains less than or equal to -0.15%, both modes are thermally distributed around zero, and correspondingly there is no visual appearance of a long-ranged wave. The critical compressive strain for this random-to-coherent rippling phase transition therefore lies between -0.15% to -0.20%, although it should be noted that this transition may be affected by the presence of the droplet and the wetting-induced curvature.

In the long-ranged coherent rippling phase, it is seen that the water droplet resides in the valley of the wave. In particular, the long-ranged wave on the graphene sheet appears to be stationary in droplet-centred coordinates, and is reflected in the time-averaged graphene heightmap $\langle h_{\mathrm{GS}}(x, y)\rangle_t$ over the droplet-centred x and y coordinates. This means that, in absolute coordinates, the motions of the long-ranged wave and the droplet are coupled tightly together, with the droplet "surfing" together with the wave in order to maintain its position in the wave valley. An example of this motion, for the sheet under -1.0% compressive strain,

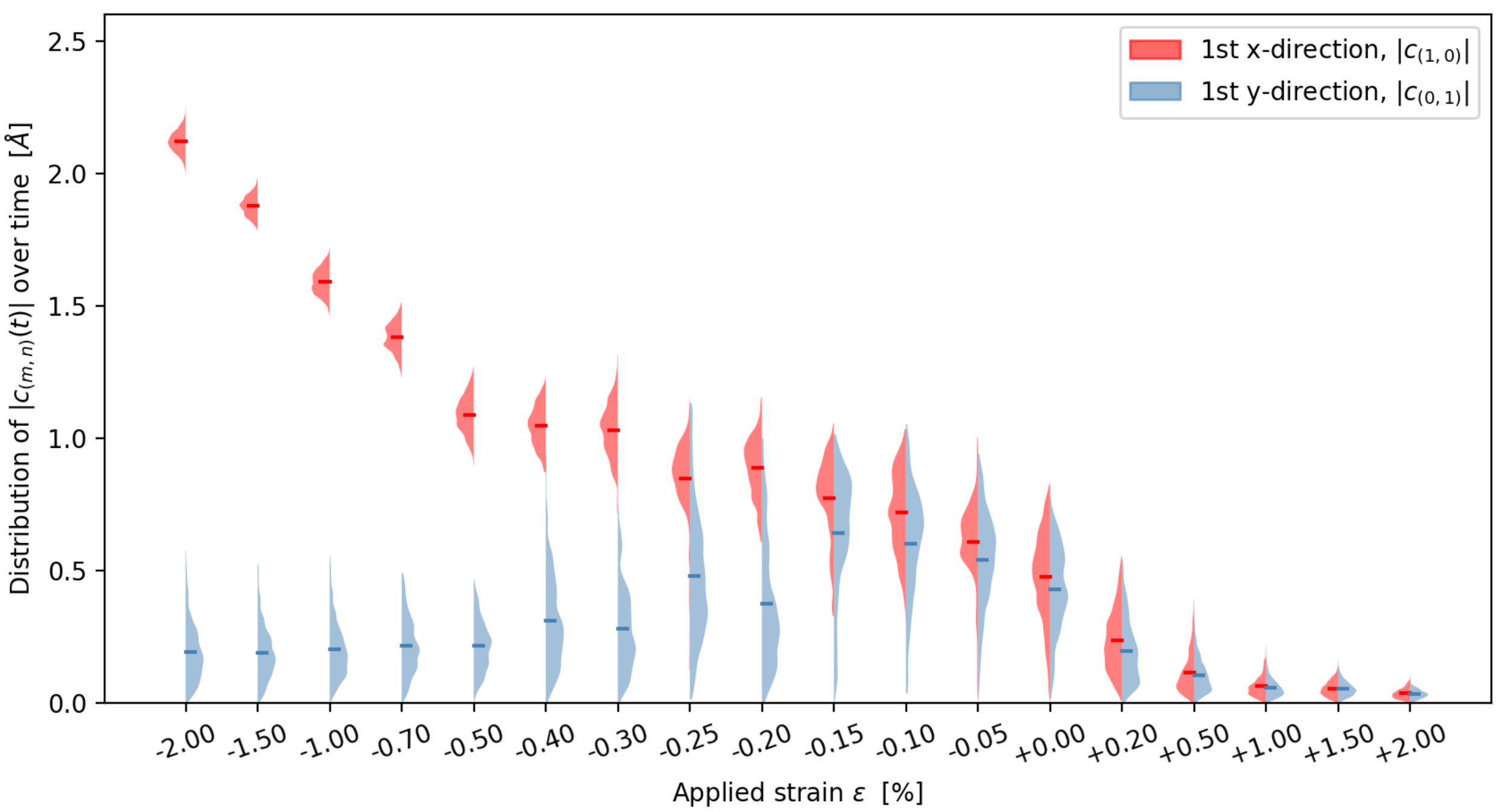

Figure S15: **The distributions of the magnitudes of the first Fourier coefficients** for the graphene heightmap $h_{\mathrm{GS}}(x, y; t)$ in the x-direction (red) and y-direction (blue) over time, for each applied strain condition. These coefficients were calculated from the simulation trajectories of the 1,000 water molecules droplet present on the graphene sheet. For compressive strains greater than or equal to -0.20%, a bifurcation forms between the dominant $(1, 0)$ mode, which gains a non-zero time-average, and the thermally distributed $(0, 1)$ mode which is centred around zero. On the other hand, for all other strain conditions, both modes are thermally distributed around zero. This indicates that the critical strain for the random-to-coherent rippling phase transition occurs between -0.15% and -0.20% compressive strain. Note that, for the purposes of clarity in plotting this graph, the x- and y-directions were swapped for some of the simulations such that the direction of spontaneous symmetry breaking for the long-ranged coherent wave is in the x-direction; in the original simulation coordinates, roughly half of the simulations with a long-ranged coherent wave had the wave occur in the y-direction (i.e. dominant $(0, 1)$ mode) instead of the x-direction.

is shown in Figure S16.

This consistent motion of the droplet together with the long-ranged coherent wave means that, relative to the droplet, the contact surface is not rotationally symmetric. Instead, one side of the droplet interface is continuously receding while the opposite side is advancing, being driven by the "surfing" motion. This results in the anisotropy of the three-phase contact line, with a range of anisotropic contact angles representing the possible dynamical contact angles between the minimum value of the receding contact angle and the maximum value of the advancing contact angle.

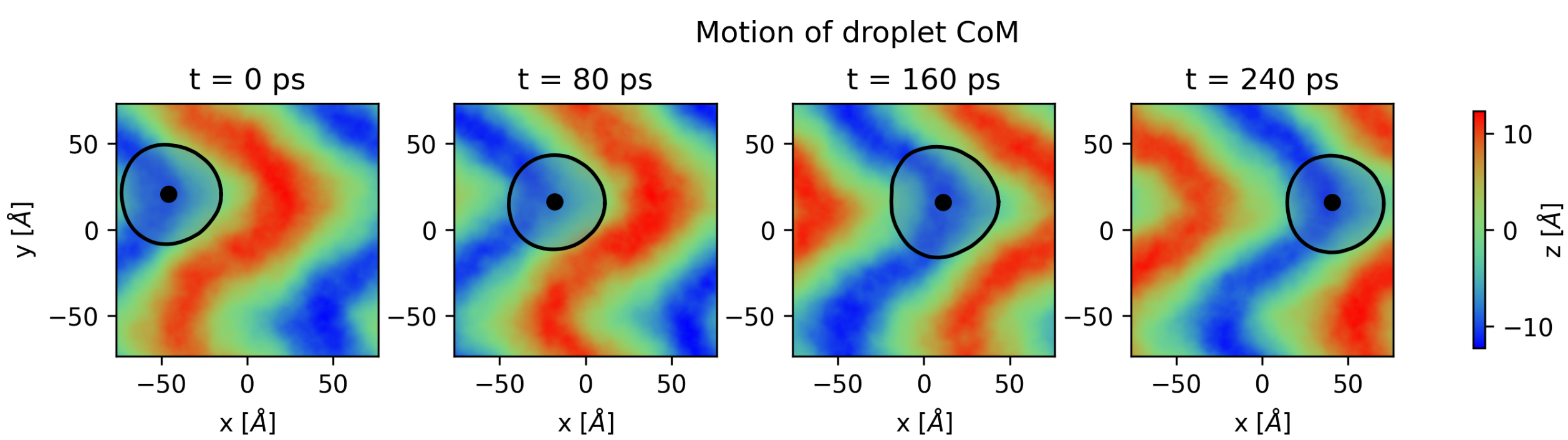

Figure S16: **The "surfing" motion of the droplet** on the long-ranged coherent rippling of the graphene sheet under compressive strain, as depicted in these series of snapshots of the xy-plane in the "raw" simulation coordinates (rather than droplet-centred coordinates). Here a droplet of 1,000 water molecules is placed upon a graphene sheet with -1.0% compressive strain. The graphene sheet is coloured according to the instantaneous z-coordinate; while the translucent blue shape indicates the location of the droplet, with the black outline showing the instantaneous three-phase contact line, and the black dot showing the droplet centre-of-mass. Across these snapshots, the long-ranged coherent wave moves from left to right, with the droplet "surfing" together with the wave in order to reside in the valley.

Droplet footprint on compressed graphene

The presence of the droplet on free-standing and tensile-strained graphene induces a persistent distortion on the graphene sheet, causing a "footprint" on the time-averaged graphene heightmap $\langle z(x,y) \rangle$ as well as a localized perturbation of the long-time inclination correlation $\mathcal{C}_{\mathrm{GS}}(\tau \to \infty)$ at the three-phase contact line. The droplet's footprint breaks the graphene sheet's translational symmetry, giving rise to both $\langle z(x,y) \rangle$ and $\mathcal{C}_{\mathrm{GS}}(\tau \to \infty)$ being perturbed from a uniform background value in a manner centred around the droplet, as shown in Figure 3 of the main text.

For compressive strains greater than or equal to -0.20%, however, this footprint no longer acts on a uniform background, as the translational symmetry of the graphene sheet is already broken by the long-ranged coherent ripple wave. A large static value of the time-averaged graphene heightmap $\langle z(x,y) \rangle$ in droplet-centred coordinates is to be expected, following the shape of the ripple wavefront, as the droplet is co-moving with the ripple wave under its "surfing" motion. As for the long-time inclination correlation $\mathcal{C}_{\mathrm{GS}}(\tau \to \infty)$, corrugation-induced stiffening suppresses thermal ripples depending on the local curvature, thus causing $\mathcal{C}_{\mathrm{GS}}(\tau \to \infty)$ to fall at the ripple wave's peaks and troughs, and to rise in between. The droplet's footprint must therefore act 'on top of' the ripple wave's effects on $\langle z(x,y) \rangle$ and $\mathcal{C}_{\mathrm{GS}}(\tau \to \infty)$.

Examples of the droplet's footprint on a few representative compressive strains, not much larger than the random-to-coherent rippling phase transition's critical strain, are shown in Figure S17. It is seen that, as the compressive strain is increased, the long-ranged coherent ripple wave becomes stronger in amplitude, resulting in an increasingly intense background variation of both $\langle z(x,y) \rangle$ and $\mathcal{C}_{\mathrm{GS}}(\tau \to \infty)$. Consequently, the droplet's localized perturbation for both observables becomes increasingly insignificant compared to the background variation, "disappearing" into the ripple wave pattern.

The continuous "disappearance" of the droplet's footprint under increasing compressive strain, in terms of its effect on $\mathcal{C}_{\mathrm{GS}}(\tau \to \infty)$ at the droplet perimeter, is reminiscent of the

similar suppression of the $\mathcal{C}_{\mathrm{GS}}(\tau \to \infty)$ 'bump' under increasing tensile strain (main text, Figure 3f). This hints at a qualitative similarity between the maximum anisotropic contact angle under compressive strain, and how it increases with applied strain, versus the isotropic contact angle under tensile strain: namely, that corrugation-induced stiffening due to the long-ranged coherent ripple wave could also suppress the thermal ripple-wetting coupling, driving graphene to become less hydrophilic. That said, a final conclusion should be taken with the caveat that the dynamic angles for the droplet "surfing" on compressed graphene do not represent equilibrium contact angles, and may not be directly comparable to the angles under tension; nonetheless, this may explain the non-monotonic trend of graphene becoming less hydrophilic under either type of applied strain, as seen in Figure 2a of the main text.

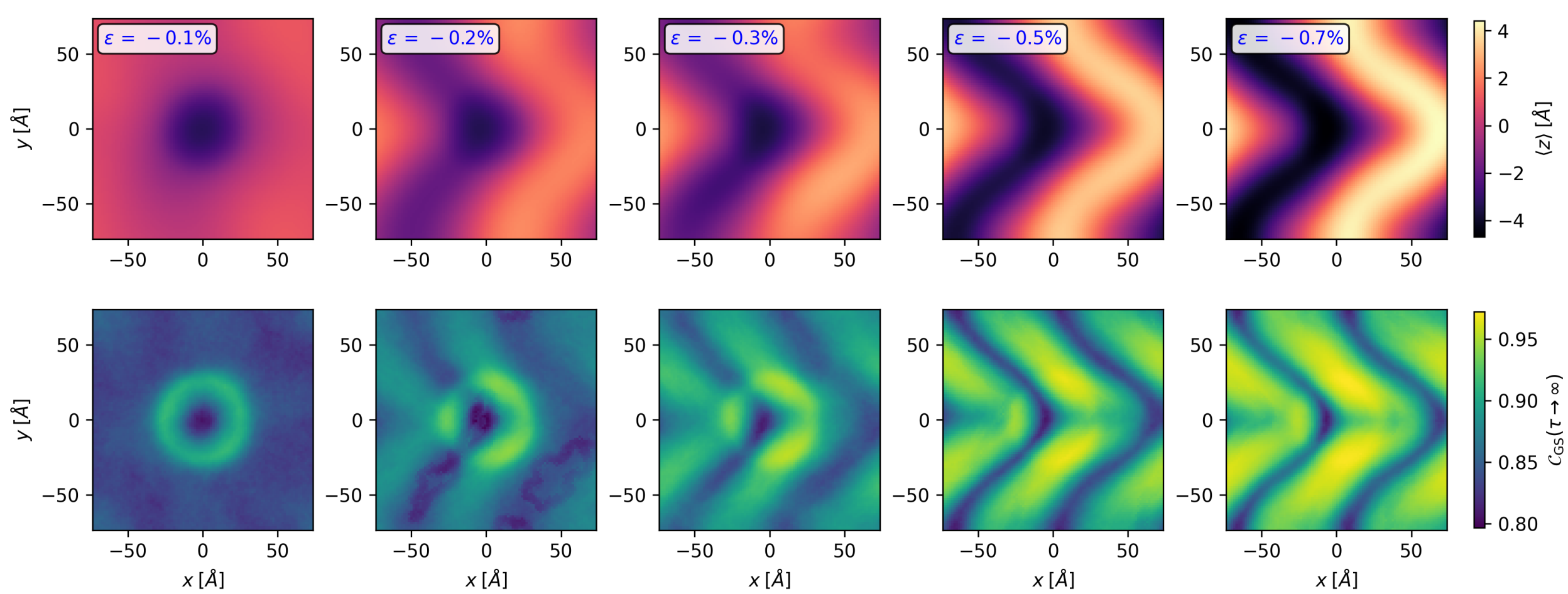

Figure S17: **The droplet's footprint on compressed graphene sheets** in droplet-centred coordinates, for compressive strains ranging from −0.10% to −0.70%. Note that the random-to-coherent rippling phase transition occurs between −0.15% to −0.20%, i.e. between the leftmost and second-leftmost columns of these plots. The top row depicts the time-averaged graphene heightmap $\langle z \rangle$, and the bottom row depicts the long-time inclination correlation $\mathcal{C}_{\mathrm{GS}}(\tau \to \infty)$. As the compressive strain is increased, the coherent ripple wave becomes stronger in amplitude, as seen in the increasingly intense "background" induced in both $\langle z \rangle$ and $\mathcal{C}_{\mathrm{GS}}(\tau \to \infty)$; consequently, while the droplet's footprint is still present, it becomes increasingly insignificant compared to the ripple wave's background.

References

(S1) Thompson, A. P.; Aktulga, H. M.; Berger, R.; Bolintineanu, D. S.; Brown, W. M.; Crozier, P. S.; in 't Veld, P. J.; Kohlmeyer, A.; Moore, S. G.; Nguyen, T. D.; Shan, R.; Stevens, M. J.; Tranchida, J.; Trott, C.; Plimpton, S. J. LAMMPS - a flexible simulation tool for particle-based materials modeling at the atomic, meso, and continuum scales. *Comput. Phys. Commun.* **2022**, *271*, 108171.

(S2) Witt, W. C. `symmetrix`. 2025; Accessed 15 Jan 2025 at https://github.com/wcwitt/symmetrix.

(S3) Castro Neto, A. H.; Guinea, F.; Peres, N. M. R.; Novoselov, K. S.; Geim, A. K. The electronic properties of graphene. *Rev. Mod. Phys.* **2009**, *81*, 109–162.

(S4) Advincula, X. R.; Litman, Y.; Fong, K. D.; Witt, W. C.; Schran, C.; Michaelides, A. How reactive is water at the nanoscale and how to control it? *Sci. Adv.* **2026**, *12*, eaeb5772.

(S5) Advincula, X. R.; Fong, K. D.; Michaelides, A.; Schran, C. Protons accumulate at the graphene-water interface. *ACS Nano* **2025**, *19*, 17728–17737.

(S6) Thiemann, F. L.; Schran, C.; Rowe, P.; Müller, E. A.; Michaelides, A. Water flow in single-wall nanotubes: oxygen makes it slip, hydrogen makes it stick. *ACS Nano* **2022**, *16*, 10775–10782.

(S7) Wei, Y.; Wang, B.; Wu, J.; Yang, R.; Dunn, M. L. Bending rigidity and Gaussian bending stiffness of single-layered graphene. *Nano Lett.* **2013**, *13*, 26–30.

(S8) Marsalek, O.; Markland, T. E. Quantum dynamics and spectroscopy of ab initio liquid water: the interplay of nuclear and electronic quantum effects. *J. Phys. Chem. Lett.* **2017**, *8*, 1545–1551.

(S9) Galib, M.; Duignan, T. T.; Misteli, Y.; Baer, M. D.; Schenter, G. K.; Hutter, J.; Mundy, C. J. Mass density fluctuations in quantum and classical descriptions of liquid water. *J. Chem. Phys.* **2017**, *146*, 244501.

(S10) Tanaka, M.; Girard, G.; Davis, R.; Peuto, A.; Bignell, N. Recommended table for the density of water between 0 °C and 40 °C based on recent experimental reports. *Metrologia* **2001**, *38*, 301–309.

(S11) Soper, A. K. Joint structure refinement of X-ray and neutron diffraction data on disordered materials: application to liquid water. *J. Phys. Condens. Matter* **2007**, *19*, 335206.

(S12) Rowlinson, J. S.; Widom, B. *Molecular theory of capillarity*; Clarendon Press, 1982; p 85–88.

(S13) Sega, M.; Fábián, B.; Jedlovszky, P. Nonzero ideal gas contribution to the surface tension of water. *J. Phys. Chem. Lett.* **2017**, *8*, 2608–2612.

(S14) Schmitz, F.; Virnau, P.; Binder, K. Logarithmic finite-size effects on interfacial free energies: phenomenological theory and Monte Carlo studies. *Phys. Rev. E Stat. Nonlin. Soft Matter Phys.* **2014**, *90*, 012128.

(S15) Schmitz, F.; Virnau, P.; Binder, K. Determination of the origin and magnitude of logarithmic finite-size effects on interfacial tension: role of interfacial fluctuations and domain breathing. *Phys. Rev. Lett.* **2014**, *112*, 125701.

(S16) Nagata, Y.; Ohto, T.; Bonn, M.; Kühne, T. D. Surface tension of ab initio liquid water at the water-air interface. *J. Chem. Phys.* **2016**, *144*, 204705.

(S17) The International Association for the Properties of Water and Steam *Revised Release on Surface Tension of Ordinary Water Substance*; 2014.

(S18) Harris, C. R.; Millman, K. J.; van der Walt, S. J.; Gommers, R.; Virtanen, P.; Cournapeau, D.; Wieser, E.; Taylor, J.; Berg, S.; Smith, N. J.; Kern, R.; Picus, M.; Hoyer, S.; van Kerkwijk, M. H.; Brett, M.; Haldane, A.; del Río, J. F.; Wiebe, M.; Peterson, P.; Gérard-Marchant, P.; Sheppard, K.; Reddy, T.; Weckesser, W.; Abbasi, H.; Gohlke, C.; Oliphant, T. E. Array programming with NumPy. *Nature* **2020**, *585*, 357–362.

(S19) Willard, A. P.; Chandler, D. Instantaneous liquid interfaces. *J. Phys. Chem. B* **2010**, *114*, 1954–1958.

(S20) Yuan, Y.; Lee, T. R. *Surface Science Techniques*; Springer series in surface sciences; Springer Berlin Heidelberg: Berlin, Heidelberg, 2013; pp 3–34.

(S21) The SciPy community CloughTocher2DInterpolator. 2025; Publication Title: SciPy v1.15.2 Manual.

(S22) Werder, T.; Walther, J. H.; Jaffe, R. L.; Halicioglu, T.; Koumoutsakos, P. On the water-carbon interaction for use in molecular dynamics simulations of graphite and carbon nanotubes. *J. Phys. Chem. B* **2003**, *107*, 1345–1352.

(S23) Liao, S.; Ke, Q.; Wei, Y.; Li, L. Water-graphene non-bonded interaction parameters: development and influence on molecular dynamics simulations. *Appl. Surf. Sci.* **2022**, *603*, 154477.

(S24) Brandenburg, J. G.; Zen, A.; Fitzner, M.; Ramberger, B.; Kresse, G.; Tsatsoulis, T.; Grüneis, A.; Michaelides, A.; Alfè, D. Physisorption of water on graphene: subchemical accuracy from many-body electronic structure methods. *J. Phys. Chem. Lett.* **2019**, *10*, 358–368.

(S25) Ma, M.; Tocci, G.; Michaelides, A.; Aeppli, G. Fast diffusion of water nanodroplets on graphene. *Nat. Mater.* **2016**, *15*, 66–71.

(S26) Meng, L.; Su, Y.; Geng, D.; Yu, G.; Liu, Y.; Dou, R.-F.; Nie, J.-C.; He, L. Hierarchy of graphene wrinkles induced by thermal strain engineering. *Appl. Phys. Lett.* **2013**, *103*, 251610.

(S27) Thiemann, F. L.; Rowe, P.; Müller, E. A.; Michaelides, A. Machine learning potential for hexagonal boron nitride applied to thermally and mechanically induced rippling. *J. Phys. Chem. C Nanomater. Interfaces* **2020**, *124*, 22278–22290.

(S28) Wang, W.; Yang, S.; Wang, A. Strain induced highly oriented graphene wrinkles. *Mater. Res. Express* **2017**, *4*, 075601.
